# Single-nucleus RNA-seq and FISH identify coordinated transcriptional activity in mammalian myofibers

**DOI:** 10.1038/s41467-020-18789-8

**Published:** 2020-10-09

**Authors:** Matthieu Dos Santos, Stéphanie Backer, Benjamin Saintpierre, Brigitte Izac, Muriel Andrieu, Franck Letourneur, Frederic Relaix, Athanassia Sotiropoulos, Pascal Maire

**Affiliations:** 1 Université de Paris, Institut Cochin, INSERM, CNRS., 75014 Paris, France; 2 Université Paris-Est Creteil, INSERM U955 IMRB., 94000 Creteil, France

**Keywords:** Biological techniques, Cell biology, Computational biology and bioinformatics, Developmental biology

## Abstract

Skeletal muscle fibers are large syncytia but it is currently unknown whether gene expression is coordinately regulated in their numerous nuclei. Here we show by snRNA-seq and snATAC-seq that slow, fast, myotendinous and neuromuscular junction myonuclei each have different transcriptional programs, associated with distinct chromatin states and combinations of transcription factors. In adult mice, identified myofiber types predominantly express either a slow or one of the three fast isoforms of Myosin heavy chain (MYH) proteins, while a small number of hybrid fibers can express more than one MYH. By snRNA-seq and FISH, we show that the majority of myonuclei within a myofiber are synchronized, coordinately expressing only one fast *Myh* isoform with a preferential panel of muscle-specific genes. Importantly, this coordination of expression occurs early during post-natal development and depends on innervation. These findings highlight a previously undefined mechanism of coordination of gene expression in a syncytium.

## Abstract (web summary)

Whether skeletal muscle fibre gene expression is coordinated as a whole in different nuclei in the fibre is unclear. Here, the authors use single nucleus RNAseq and ATACseq to show the transcriptome heterogeneity of muscle nuclei in the adult mouse fibre, with correlations between the two datasets.

## Introduction

Skeletal muscles constitute 40 to 50% of our body mass and are principally composed of very long myofibers attached to bones via tendons, innervated by motoneurons, and surrounded by vessels and fibroblasts cells. The myofibers themselves are syncytia composed of hundreds of post-mitotic nuclei sharing the same cytoplasm, generated by the fusion of myoblasts during development^1^. However, the protein content of myofibers is not homogenous along their length. The proteins required for the formation of the neuromuscular junction (NMJ) accumulate in the center of the myofiber whereas specific proteins for myotendinous junction specialization (MTJ) accumulates at the periphery of the fiber. Two mechanisms could account for this regionalization: the transport of specific proteins to these sites^2^, and the specialization of transcription within the nuclei in these sites^3,4^. At the NMJ, specific myonuclei express *AchR* and *AchE*^5^ and at the MTJ-specific myonuclei express *Col22a1*^6,7^. However, the precise genetic program operating in these specific myonuclei and the transcription factors involved in this specialization during development, compared to the other myonuclei (called body myonuclei), is far from being understood.

In body myonuclei that constitute the vast majority of myonuclei in the fiber, heterogeneity in nuclear protein import^8^ and stochastic gene expression^9^ have been proposed to occur, suggesting that body myonuclei along the myofiber are not equivalent. Accordingly, gene transcription could occur stochastically (bursts of transcription), resulting in apparent uncoordinated gene expression at a given locus between the nuclei of the same fiber. Furthermore, in other syncytia such as osteoclasts and syncytiotrophoblasts, transcriptional activity is heterogeneous, varying from one nucleus to the other, indicating that their nuclei, although they share the same cytoplasm, are not coordinated^10,11^. Whether the hundreds of body nuclei along the myofiber are transcriptionally active at the same time and express the same set of genes remains debated.

Adult skeletal muscles are composed mainly of slow and fast myofibers and are usually classified by their MYH (myosin heavy chain) expression profile^12–15^. MYH is the primary determinant of the efficiency of muscle contraction: slow myofibers in mice express *Myh7* (*MyHC-I*), and fast myofibers express *Myh2* (*MyHC-IIA*), *Myh1* (*MyHC-IIX*) or *Myh4* (*MyHC-IIB*). These different *Myh* isoforms are coded by different genes, and in mammals, fast *Myh (*f*Myh)* genes, including *Myh2*, *Myh1*, *Myh4*, embryonic *Myh3*, neonatal *Myh8*, and extraocular *Myh13* are clustered on a single locus while the slow *Myh7* gene lies on another locus^16^. Myofibers can express one, two or three MYH isoforms at the same time at protein and mRNA level^17–20^. Myofibers expressing more than one MYH isoform are called hybrid fibers in contrast to pure fibers expressing only one isoform. Post-transcriptional mechanisms involving antisense RNA^21^ or mir-RNA^22^ may participate in the control of the accumulation of specific MYH proteins in both homogenous and hybrid myofibers. Alternatively, hybrid fibers may be constituted of regionalized *Myh* genes expression or of uniform *Myh* genes co-expression along the fiber. For the well-studied *ß-Globin* locus whose organization is reminiscent of the f*Myh* locus, it was shown that the Locus Control Region (LCR) establishes contacts and activates a single gene at the locus at a given time^23^. It was more recently demonstrated that two adjacent genes can be expressed in the same nucleus at a given time by a shared enhancer^24^. Whether one myonucleus can activate one, two or three f*Myh* genes at the same time remains to be established.

In this report, we sequence the RNAs and the chromatin accessibility from single nucleus (snRNA-seq and snATAC-seq) isolated from adult skeletal muscles to characterize the transcription profile of known cell populations present in adult skeletal muscles, including Fibro-adipogenic progenitors (FAPs), tenocytes, myogenic stem cells and myonuclei, establishing a blueprint of the transcriptional activity of all its nuclei. We further identify three main populations of myonuclei: NMJ, MTJ, and body myonuclei with specific transcriptional and chromatin accessibility. Our results show that most myonuclei express only one f*Myh* gene at the same time. By visualizing in vivo in mechanically isolated myofibers the localization of f*Myh* pre-mRNAs, we validate these results showing a coordination of f*Myh* expression in the myonuclei of the majority of myofibers of the fast extensor digitorum longus (EDL). We also identify a minority of hybrid myofibers with nuclei expressing several f*Myh* isoforms at the same time. Those hybrid myofibers are more abundant in slow soleus muscle and during denervation in the fast EDL. Last, we characterize how adult f*Myh* genes become activated during post-natal development and show the importance of innervation for their coordination.

## Results

### SnRNA-seq identifies several cell populations in adult skeletal muscles

**Fig. 1:**
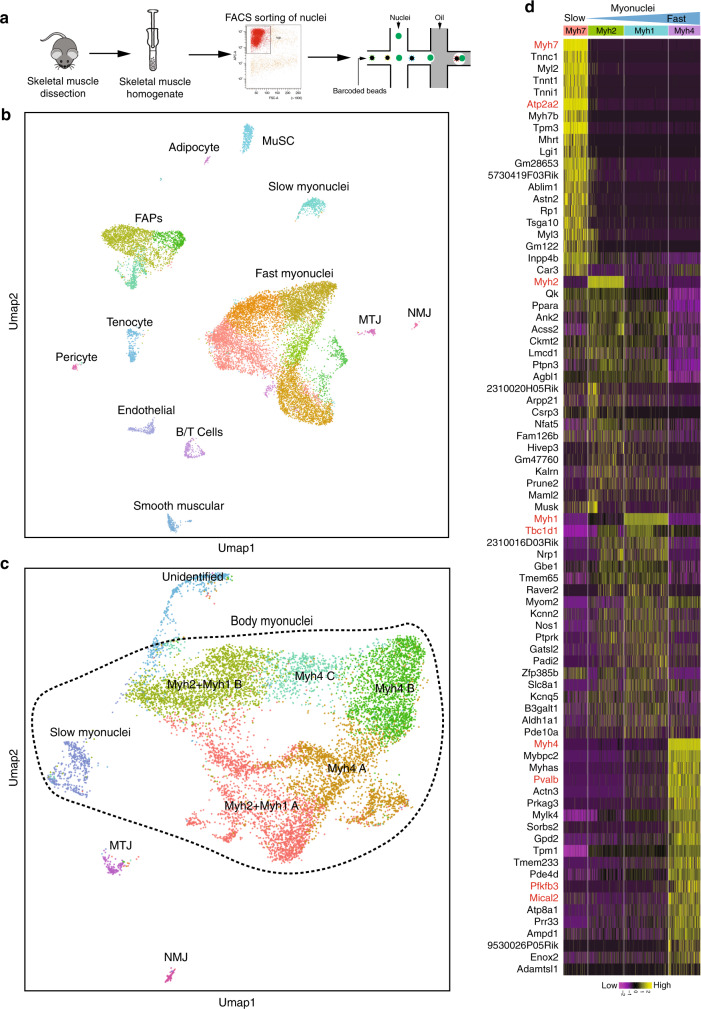
SnRNA-seq analysis from adult slow and fast skeletal muscles. **a** Graphical scheme of the experiments used for snRNA-seq analysis of adult skeletal muscles. **b** Uniform Manifold Approximation and Projection (Umap) diagram from snRNA-seq from adult skeletal muscle. MuSC skeletal muscle stem cells, FAPs fibro-adipogenic progenitors, MTJ myotendinous junction, NMJ neuromuscular junction. **c** Umap diagram of snRNA-seq data with myonuclei only. Myonuclei are separated into five clusters: slow, fast, MTJ, NMJ, and unidentified myonuclei. The slow and fast myonuclei are called body nuclei and are surrounded by dotted lines. Five subcategories of fast myonuclei are clustered. **d** Heatmap of genes upregulated (yellow) and downregulated (violet) in the myonuclei expressing the different slow and fast *Myh* isoforms. The values correspond to *z*-scores of normalized counts.

**Fig. 2:**
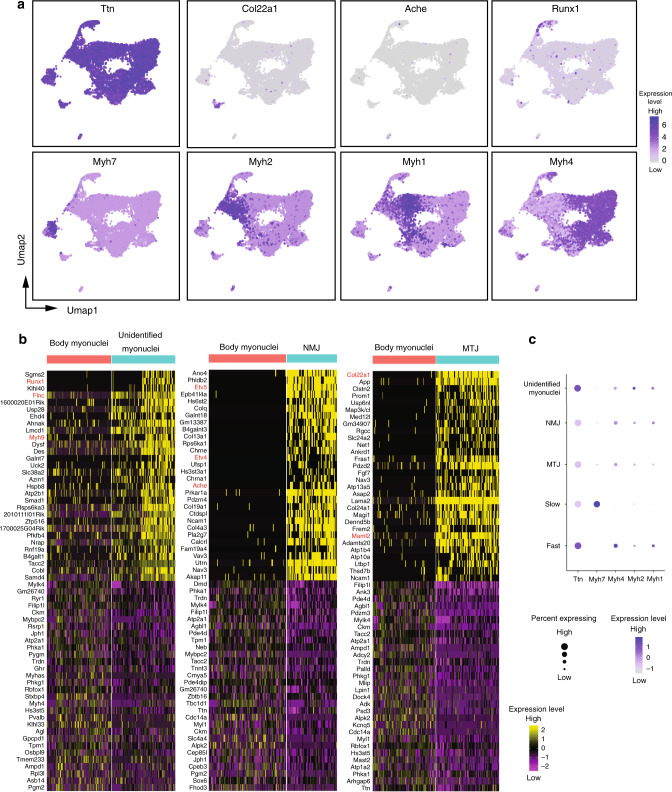
The different populations of myonuclei. **a** Umap Plots showing the expression of several markers used to identify the different types of myonuclei. The intensity of the blue color reflects the level of expression of the gene. **b** Heatmap of the top 30 genes upregulated and downregulated (yellow) in body myonuclei versus unidentified myonuclei (left), in body myonuclei versus NMJ nuclei (center), and body myonuclei versus NMJ nuclei (right). The values correspond to *z*-scores of normalized counts. **c** Dot-plots from snRNA-seq experiments showing the expression of the slow and fast *Myh* in the different populations of myonuclei. NMJ and MTJ nuclei expressed *Myh* like other myonuclei.

To characterize the transcriptional profile of myonuclei within myofibers, we developed single nucleus RNA-seq (snRNA-seq) experiments using a droplet-based platform on purified nuclei from different skeletal muscles of adult mice (Fig. 1a and Supplementary Fig. 1a–d). By using Seurat software^25^ and Uniform Manifold Approximation and Projection (UMAP) clustering we were able to detect a total of 19 different cell populations clusters present in the different muscles (Fig. 1b and Supplementary Fig. 2b, c). The origin of the nuclei was identified by specific markers (Fig. 1b and Supplementary Figs. 1e and 2a, Supplementary Data 1), with 68% of nuclei originating from myofibers, 19% from FAPs, and around 13% from other cell types, including tenocytes, endothelial and lymphoid cells, smooth muscle cells, and myogenic stem cells (Supplementary Fig. 2d). To look at the heterogeneity of the myonuclei, we have then reclustered the myonuclei expressing *Titin* (*Ttn)* gene to remove all the non-fiber nuclei (Fig. 1c). Several populations of myonuclei where identified, among which MTJ myonuclei (expressing *Col22a1*), NMJ myonuclei (expressing *Ache*), a yet unknown myonuclei subpopulation (expressing *Myh9*, *Flnc*, *Runx1)*, and body myonuclei accounting for 94% of all myonuclei (Figs. 1c and 2a). By comparing gene expression in body nuclei and specialized nuclei, we characterized numerous known (*Ache* and *Etv5* for NMJ^5^; *Coll22a1* for MTJ^6^ and unknown genes specific to these specialized myonuclei (*Etv4* for NMJ, *Maml2* for MTJ) (Fig. 2b). Unidentified, NMJ and MTJ myonuclei also expressed *Myh* genes (Fig. 2c). Slow myonuclei expressing *Myh7* clustered independently from the fast *Myh1/2/4* myonuclei. The fast myonuclei formed a large cluster that could be subdivided into several subpopulations, all expressing sarcomeric genes like *Ttn* and various *Myh* isoforms (Figs. 1c and 2a). *Myh4* + myonuclei represented 51% of this fast myonuclei population. We also compared the expression of genes in slow and fast nuclei expressing the different types of *Myh* (Fig. 1d). Fast *Myh2* and *Myh1* nuclei showed a similar transcription program, contrasting with slow *Myh7* and very fast *Myh4* nuclei each showing a distinct transcriptional program (Fig. 1d). Interestingly, three main categories of *Myh4* nuclei were identified, identifying transcriptional heterogeneity among these nuclei (Supplementary Fig. 3a–c). We observed higher expression of *Mical2*, *Pde4d* and *Sorbs2* in the *Myh4B* myonuclei subgroup, of *Taco1*, *Neat1* and of *Kpna1* in *Myh4A* myonuclei subgroup, and of *Esrrγ*, *Agbl1* and *Ptpn3* in *Myh4C* myonuclei subgroup. These *Myh4* nuclei subpopulations were distributed differently in distinct muscles, with a higher percentage of the *Myh4C* population in the fast Tibialis compared to the fast Quadriceps and a higher percentage of *Myh4B* nuclei in the Quadriceps as compared with the Tibialis (Supplementary Fig. 3c). From these experiments, we concluded that *Myh4* nuclei can have different transcription profiles, depending on the muscle, what was presently unsuspected.

### SnATAC-seq identifies heterogenous transcriptional programs in myonuclei

**Fig. 3:**
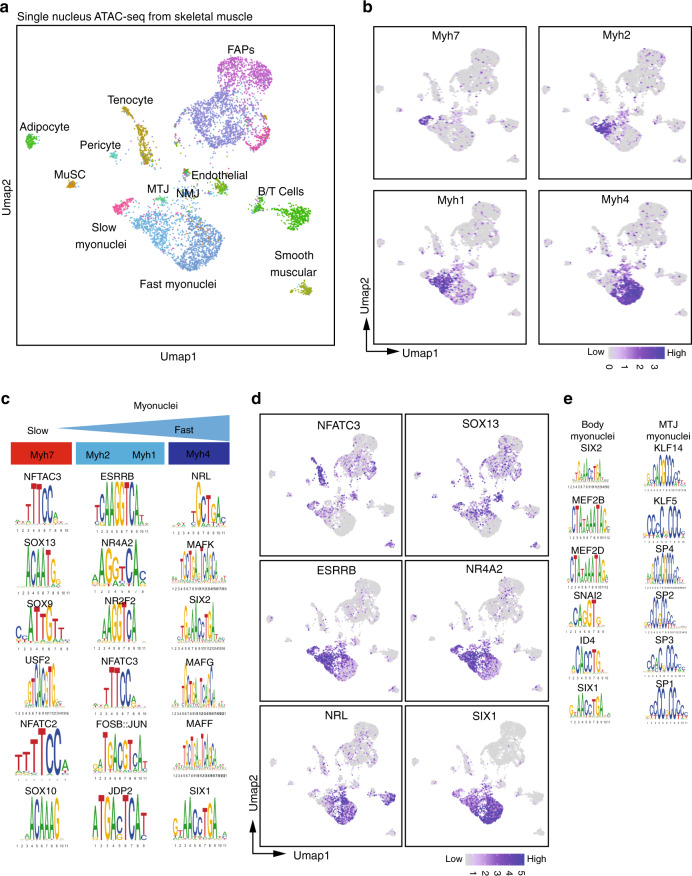
SnATAC-seq analysis from adult slow and fast skeletal muscles. **a** Umap Plots from snATAC-seq from adult skeletal muscle based on chromatin accessibility. MuSC skeletal muscle stem cells, FAPs fibro-adipogenic progenitors, MTJ myotendinous junction, NMJ neuromuscular junction. **b** Umap plots showing the chromatin opening of slow and fast *Myh* genes used to identify the different types of myonuclei. The intensity of the blue color reflects the level of chromatin accessibility of the gene. **c** Position weight matrix (PWM) motifs showing the enrichment of TF motifs in differential peaks in the myonuclei expressing the different *Myh* genes. **d** Umap Plots showing the motif activity of TF in the different types of myonuclei. The intensity of the blue color reflects the level of TF motif enrichment in the nuclei. **e** PWM motif showing the enrichment of TF motifs in differential peaks in the body and MTJ myonuclei.

Recent studies profiled the genome-wide chromatin accessibility of adult skeletal muscles^26^. However, this global approach did not discriminate the chromatin status of each cell population present in skeletal muscles. Thus, we set up a protocol to perform single nucleus ATAC-seq (snATAC-seq) with purified nuclei from adult soleus and quadriceps using a droplet-based platform. Briefly, nuclei were transposed using Tn5 transposase and then encapsulated into gel beads emulsion using 10X protocol. After quality assessments and filtering (Supplementary Fig. 4a–c), 132 966 peaks were identified from 6037 sequenced nuclei. To identify the different populations of nuclei, we classified nuclei from ATAC-seq based on snRNA-seq experiments from Fig. 1b: we used methods for cross-modality integration of snRNA-seq and snATAC-seq data and label transfer from Signac software (Fig. 3a and Supplementary Fig. 4d, e). This classification was robust, as shown by the chromatin accessibility of the *Myh* genes present only in myonuclei (Fig. 3b and Supplementary Fig. 5a, b). Using chromVar we characterized the transcription factors (TF) variations in motif accessibility between the different types of myonuclei (Fig. 3c). Knowing that this analysis frequently does not distinguish between related TFs of the same family (usually sharing similar motifs)^27^, we could characterize that the NFAT and SOX binding motifs accessibility are enriched in slow *Myh7* myonuclei, ESRRβ and NR4A2 motifs in fast *Myh2* and *Myh1* myonuclei and SIX and NRL/MAF motifs in fast *Myh4* myonuclei (Fig. 3c, d and Supplementary Fig. 3b). These distinct combinations of TF could participate in the genetic code controlling the expression of specific fast-subtypes and slow sarcomeric genes and of genes associated with myofiber specialization (Fig. 1d). Equivalently to Fig. 1d presenting genes differently expressed in slow and fast myonuclei (from snRNA-seq data), we observed specific chromatin accessibility between slow and fast myonuclei in genes differently expressed in slow and fast myonuclei. For exemple, the gene *Pvalb* expressed only in fast *Myh4* myonuclei (Fig. 1d) presented a strong chromatin accessibility only in fast *Myh4* myonuclei (Supplementary Fig. 5d). At the opposite a strong chromatin accessibility in the *Atp2a2* was observed in *Myh7* nuclei only where this gene is expressed (Fig. 1d and Supplementary Fig. 5e). These results showed with a high-resolution how slow and fast-subtypes genetic programs are associated with different sets of TF controlling the expression and the chromatin accessibility of a set of co-expressed genes. Interestingly, a specific set of TF motifs is enriched in MTJ myonuclei as compared to body myonuclei, suggesting their participation in regionalized expression of specific genes such as *Col22a1* (Fig. 3e and Supplementary Fig. 5c).

### Most myonuclei express a single f*Myh* gene

**Fig. 4:**
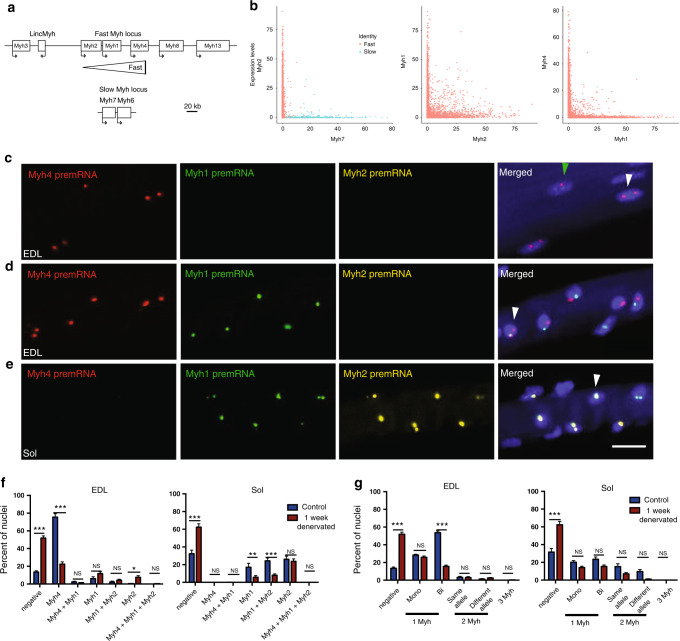
The majority of myonuclei express only one isoform of *Myh*. **a** Diagram of the mouse *Myh* fast and slow loci. **b** Analysis of *Myh* isoforms expression in myonuclei from snRNA-seq data. Each dot corresponds to a myonucleus and the *x*- and *y*-axis corresponds to the indicated *Myh* expression level. **c** RNAscope experiments on isolated fibers from EDL to visualize fast *Myh4* (red), *Myh1* (green) and *Myh2* (yellow) pre-mRNAs. *Myh* pre-mRNA can be detected as two transcribed alleles (white arrowhead) or as a single allele (green arrowhead). **d** Same as **c** showing nuclei expressing at the same time different isoforms of *Myh* from each *Myh* allele and nuclei co-expressing (arrowhead) at the same time two isoforms of *Myh* from the same allele. **e** Same as **c** in soleus showing nuclei co-expressing at the same time *Myh1* and *Myh2* pre-mRNAs from each allele (arrowhead). **f** Percentage of nuclei expressing f*Myh* pre-mRNAs, in control and 1 week after denervation, in fast myofibers from EDL and soleus. **g** Percentage of nuclei expressing one, two, and three isoforms of pre-mRNAs of *Myh* in control or 1-week denervated EDL and soleus. Percentage of mono- and bi-allelic expression is also indicated. For **c**–**e** scale bar: 20 μm. For **f** and **g**, *n* = 3 and 20 fibers per animal. Numerical data are presented as mean ± s.e.m. *P < 0.05, **P < 0.01, ***P < 0.001. Source data for (**f**, **g**) are provided in the Source Data file.

**Fig. 5:**
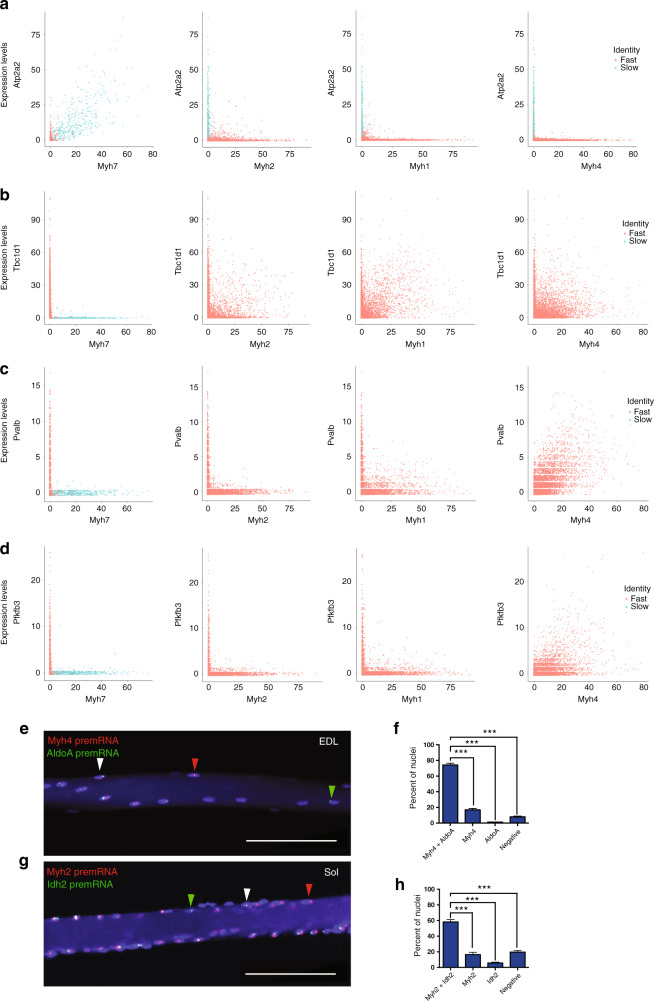
Sarcomeric and metabolic genes are co-expressed in myonuclei. **a** Analysis of *Atp2a2* and *Myh* isoform expression in myonuclei from snRNA-seq data from Fig. 1a. Each dot corresponds to one myonucleus and the axis correspond to the gene expression level. *Atp2a2* is expressed only in most *Myh7* and in few *Myh2* nuclei. **b** Same as **a** with *Tbc1d1* expression. *Tbc1d1* is expressed only in fast and not in slow nuclei. **c** Same as **a** with *Pvalb* expression. *Pvalb* is expressed only in *Myh1* and *Myh4* nuclei. **d** Same as **a** with *Pfkfb3* expression. *Pfkfb3* is expressed preferentialy in *Myh4* nuclei. **e** RNAscope on isolated fibers from EDL showing the localization of *Myh4* (red) and *AldoA* (green) pre-mRNAs. Most myonuclei expressed at the same time *Myh4* and *AldoA* (white arrowhead). However, some nuclei expressed *Myh4* without *AldoA* (red arrowhead), and some nuclei are negative for both genes. **f** Percentage of the nuclei co-expressing *Myh4* and *AldoA* or expressing only *Myh4* or *AldoA* and negative nuclei in EDL fibers. **g** RNAscope on isolated fibers from soleus showing the localization of *Myh2* (red) and *Idh2* (green) pre-mRNAs. Like in **e** most myonuclei expressed at the same time *Myh2* and *Idh2* (white arrowhead). However, some nuclei expressed *Myh2* without *Idh2* (red arrowhead) and other nuclei are negative for both genes. **h** Percentage of the nuclei co-expressing *Myh2* and *Idh2* or expressing only *Myh2* or *Idh2* and negative nuclei in soleus fibers. For e.g., scale bar: 100 μm. For **f**, **h** the graphs show data pooled from three animals and fifteen fibers per animal. Numerical data are presented as mean ± s.e.m. *P < 0.05, **P < 0.01, ***P < 0.001. Source data for **f**, **h** are provided in the Source Data file.

Fast *Myh* genes are located side by side on the same locus and it is presently unknown whether they can be transcribed simultaneously (Fig. 4a). Using snRNA-seq, we found that only rare mixed nuclei co-expressed simultaneously *Myh7* and *Myh2* or *Myh1* and *Myh4* (Fig. 4b). Mixed nuclei co-expressing *Myh2* and *Myh1* were more numerous. Other combinations of co-expression were not detected (Supplementary Fig. 6a). This showed that the vast majority of adult myonuclei analyzed were pure and expressed only one *Myh* gene. To visualize the expression of f*Myh* genes, we performed fluorescent in situ hybridization (RNAscope) experiments using intronic probes to detect f*Myh* pre-mRNA in myofibers from EDL and soleus muscles (Fig. 4c–e). Myonuclei expressed f*Myh* genes in a mono or bi-allelic manner (Fig. 4c). Expression of two distinct f*Myh* genes from identical or distinct alleles was detected in some nuclei (Fig. 4d). Expression of two f*Myh* genes by the same allele (single bicolor dot) indicated that two *Myh* genes were transcribed simultaneously on the same locus (Fig. 4e–g). In some myonuclei, no f*Myh* pre-mRNA was detected. Quantification of RNAscope experiments is provided in (Fig. 4f, g): in EDL, the majority (>80%) of nuclei expressed only one *Myh* isoform and <5% co-expressed two isoforms. The number of nuclei co-expressing two isoforms of *Myh* was highest in the soleus, where ~25% co-expressed *Myh1* and *Myh2* (Fig. 4f). The co-expression was in most cases from the same allele (Fig. 4g), suggesting that *Myh2* and *Myh1* can be activated simultaneously from one allele. The results of RNAscope experiments thus confirmed snRNA-seq results, showing that almost all myonuclei were pure, expressing only one *Myh* isoform at a time, except in the soleus. Then we tested whether different populations of myonuclei express either metabolic, calcium handling or sarcomeric genes, or if all myonuclei co-express simultaneously these different categories of genes. SnRNA-seq data showed that most *Myh4* myonuclei expressed the calcium handling gene *Pvalb* and the glycolytic gene *Pfkfb3*, that a majority of f*Myh* myonuclei expressed *Tbc1d1* involved in muscle glucose uptake, and most *Myh7* nuclei expressed the sarcoplasmic reticulum Calcium transporting 2 gene *Atp2a2* (Fig. 5a–d). These results were confirmed by RNAscope visualizing the localization of the pre-mRNA of *AldolaseA*, associated with glycolytic metabolism in myofiber^28^, and of *Idh2*, associated with oxidative metabolism^29^. Accordingly, more than 70% of *Myh4* expressing nuclei also expressed the glycolytic enzyme *AldolaseA* pre-mRNA, while the TCA cycle isocitrate dehydrogenase *Idh2* pre-mRNA was undetectable. At the opposite most *Myh2* expressing nuclei expressed *Idh2* pre-mRNA (Fig. 5e–h), while *AldolaseA* pre-mRNA was not detected. Thus, myonuclei co-expressed coordinated specialized sarcomeric, calcium handling and metabolic genes. Altogether these results showed that f*Myh* gene expression, and more generally muscle gene expression within the myofiber syncytium is not random but precisely controlled, each myonucleus showing a precise pattern of co-expressed genes.

### Transcription is coordinated in myonuclei along the myofiber

**Fig. 6:**
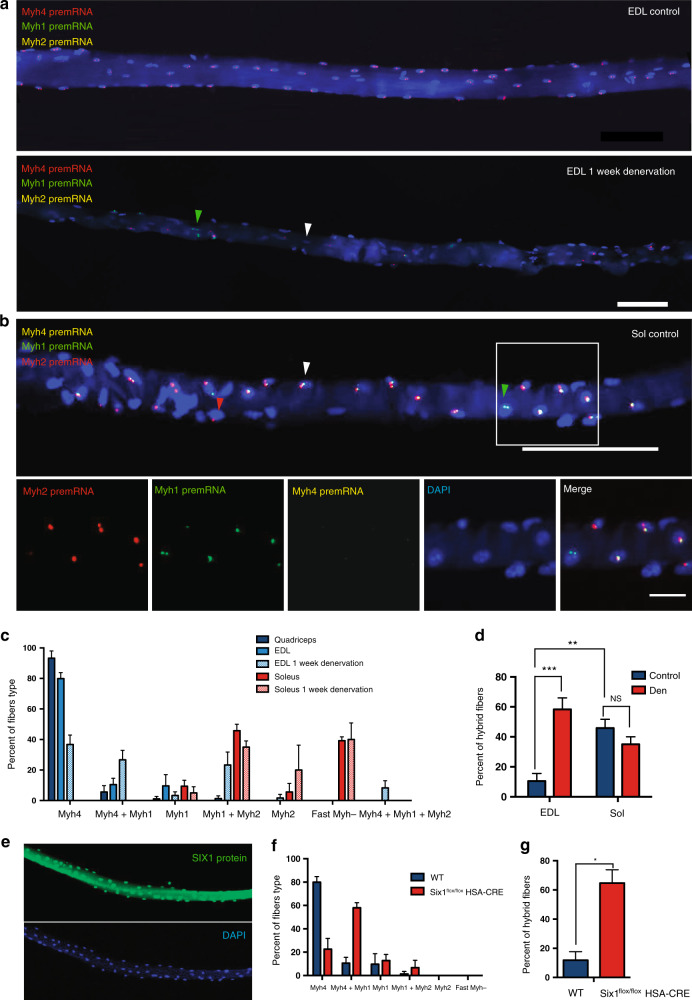
Coordination of fast *Myh* expression along myofibers. **a** RNAscope on isolated fibers from EDL showing the localization of *Myh4* (red), *Myh1* (green), and *Myh2* (yellow) pre-mRNAs. Up: EDL myofiber showing coordination of *Myh4* expression between nuclei (pure myofiber). Down: EDL myofiber after 1 week of denervation. After denervation, myonuclei are no more coordinated in the myofiber and express either *Myh4* or *Myh1* (green arrowhead) or co-express both isoforms (hybrid myofiber). In most myonuclei, f*Myh* pre-mRNAs are no more detected (white arrowhead). **b** Same as **a** with *Myh4* (yellow), *Myh1* (green), and *Myh2* (red) pre-mRNAs in a soleus hybrid myofiber. The white arrowhead shows a mixed *Myh1, Myh2* myonucleus, whereas the red arrowhead shows a pure *Myh2* myonucleus. **c** Percentage of the different types of fibers observed in quadriceps, EDL and soleus, and 1 week after denervation. **d** Percentage of hybrid (de-coordinated) fibers in control EDL and soleus and after 1 week of denervation. **e** SIX1 accumulates in all myonuclei of adult EDL fibers as identified by immunohistochemistry with SIX1-specific antibodies. **f** Percentage of the different types of EDL fibers observed in WT and *Six1*^*flox/flox*^*;HSA-CRE* mice by RNAscope experiments. **g** Percentage of hybrid EDL fibers (de-coordinated) in WT and *Six1*^*flox/flox*^*;HSA-CRE* mice. For **a**, **b** (up) scale bar: 100 μm. For **b** (down) scale bar: 20 μm. For **c**, **d**, *n* = 3 and 20 fibers per animal. For **f**, **g** the graphs show data pooled from three animals and more than fifty fibers in total. Numerical data are presented as mean ± s.e.m. *P < 0.05, **P < 0.01, ***P < 0.001. Source data for **c**, **d**, **f**, **g** are provided in the Source Data file.

We next evaluated the coordination of f*Myh* genes expression in distinct myonuclei of the same fiber. In EDL, coordinated *Myh* expression was detected in all myonuclei in a majority of fibers (Fig. 6a, upper panel). In contrast, a large number of uncoordinated fibers was detected in soleus (Fig. 6b). Different nuclei within the same soleus fiber were detected expressing different f*Myh* isoforms, while some nuclei co-expressed two isoforms. Quantification of these experiments is provided in (Fig. 6c, d). We never observed hybrid coordinated myofibers: meaning that we did not observe that all nuclei of a given fiber express the same combination of 2 or 3 *Myh* genes. In quadriceps and EDL, most myofibers (90%) showed *Myh* gene expression coordination between their myonuclei expressing only *Myh4* (Fig. 6c, d). *Myh4* mRNA accumulated homogenously in the coordinated fibers expressing *Myh4* pre-mRNA (Supplementary Fig. 7a). Interestingly, half of the fibers from the soleus were hybrid (Fig. 6c, d). In hybrid *Myh4* and *Myh1* fibers, *Myh4* mRNA accumulated less efficiently, and did not spread far from the nuclei expressing *Myh4* pre-mRNA (Supplementary Fig. 7a), demonstrating that mRNA diffusion was confined within myonuclear domains^8^. The transcriptional program of *Myh4*-positive myonuclei may differ in hybrid myofibers from pure myofibers, explaining the heterogeneity of *Myh4* myonuclei observed by snRNA-seq (Supplementary Fig. 3b, c). *Myh4* pre-mRNA was detected also in NMJ myonuclei, as identified by *AchE* mRNA expression (Supplementary Fig. 7b).

### Innervation controls myonuclei coordination along the myofiber

To understand the basis behind pure and mixed myonuclei and their coordination along the myofiber, we tested whether innervation, known to modulate adult muscle fiber type^14,15,30^, was involved in the coordination process. At the protein level, 1 week after denervation, we observed a preferential atrophy of fast MYH4 and MYH1-positive fibers and 4 weeks after denervation a fast to slow transition in all muscles (Supplementary Fig. 8a). In agreement, 1 week after denervation, the number of nuclei negative for fast *Myh* genes was increased (Figs. 4f and 6a). The percentage of uncoordinated myofibers after 1-week denervation in the EDL was increased more than five-fold but did not significantly differ in soleus (Fig. 6d). These observations showed that innervation is absolutely required to coordinate fast *Myh* genes expression in the nuclei within myofibers in EDL (Fig. 6a–d and Supplementary Fig. 8d). Shown in Supplementary Fig. 8e, a strong increase of *Myh2* and *Myh1* pre-mRNAs was detected in the MTJ myonuclei of EDL 1 week after denervation, suggesting that in absence of innervation or muscle contraction, MTJ myonuclei expressed a distinct *Myh* gene than body myonuclei^31^. These data support an important role of innervation in the activation of f*Myh* genes transcription and coordination in myonuclei along the myofiber. Altogether these experiments identified that in normal conditions myonuclei of fast myofibers (Quadriceps and EDL) are coordinated and that denervation induced an increased number of de-coordinated hybrid fibers associated with a shift of *Myh* expression toward slower isoform. In the soleus more hybrid fibers are present, and denervation did not induce drastic changes of f*Myh* expression like the ones observed in fast muscles. This suggests that the expression of *Myh1* and *Myh2* in the soleus is less under the control of innervation than the expression of *Myh4* in fast muscles, according to published data^32–34^. Knowing that in adult myofibers the homeoprotein SIX1 controls the expression of many fast/glycolytic muscle genes among which f*Myh* genes^35,36^ and that SIX binding motifs accessibility are enriched in *Myh4* myonuclei (ref. ^26^ and Fig. 3c), we looked at the nuclear accumulation of this protein in fast myonuclei along EDL myofibers. As shown in Fig. 6e we observed robust SIX1 accumulation in all myonuclei from an extremity to the other of the fiber. To further test the involvement of SIX1 in f*Myh* gene coordination along the myofiber we tested the consequences of its absence in mutant myofibers from muscle-specific *Six1* conditional knock-out (Fig. 6f, g). In absence of *Six1*, EDL myofibers appeared slower and presented an increased number of non-coordinated hybrid fibers (Fig. 6f, g), showing that SIX transcription complexes participate to coordinate the expression of f*Myh* genes in adult myonuclei.

### Coordination of myonuclei during development

**Fig. 7:**
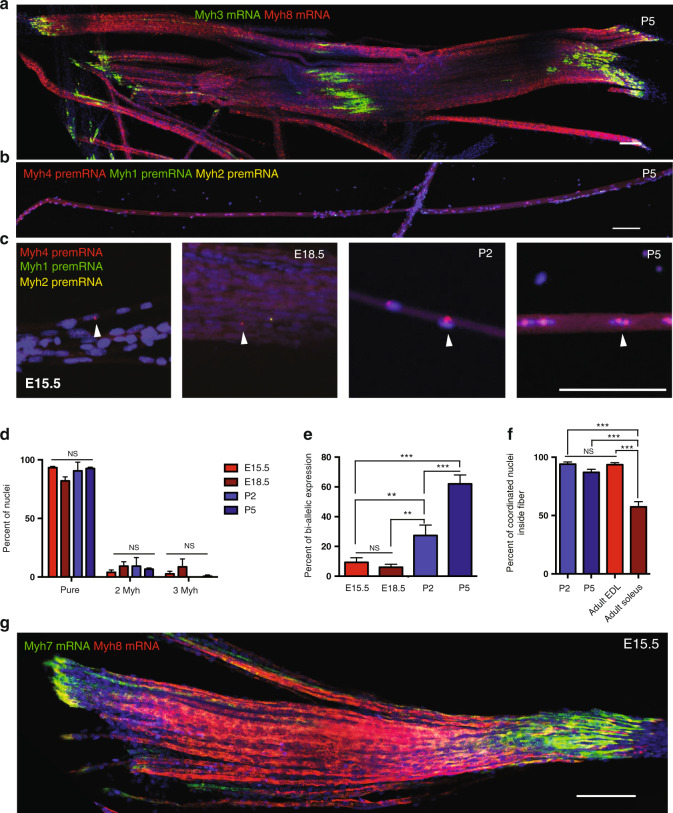
f*Myh* expression during development is regionalized. **a** RNAscope against *Myh3* (green) and *Myh8* (red) mRNA on isolated fibers from forelimbs at 5 days post-natal (P5). The MTJ and NMJ areas showed an accumulation of *Myh3* mRNA. **b** RNAscope against *Myh4* (red), *Myh1* (green), and *Myh2* (yellow) pre-mRNAs on isolated fibers at E15.5, E18.5, 2 (P2), and 5 (P5) days post-natal. **c** Same as **b**. Zoom showing the increase of adult fast *Myh*-positive (arrowhead) nuclei after birth. **d** Percentage of nuclei expressing one, two, or three adult f*Myh* isoforms at the same time at different developmental stages. **e** Percentage of nuclei with bi-allelic expression of *Myh* genes during development. **f** Percentage of coordinated nuclei inside myofibers at different developmental stages. **g** RNAscope against *Myh7* (green) and *Myh8* (red) mRNAs on isolated fibers at E15.5. *Myh7* mRNAs accumulate in MTJ areas of all myofibers at E15.5. For (**a**–**c**, **g**) scale bar: 100 μm. For **d**, *n* = 3 and 50 nuclei per animal. For **e**, **f** *n* = 3 and 20 fibers per animal. Numerical data are presented as mean ± s.e.m. *P < 0.05, **P < 0.01, ***P < 0.001. For **a** and **g** representative RNAscope experiments are presented, *N* = 3. Source data for **d**–**f** are provided in the Source Data file.

We next addressed how f*Myh* expression coordination appears during development. A sequential transition from embryonic to neonatal and to adult myosin heavy chain isoforms has already been characterized in skeletal muscles during mouse post-natal development^37^. We further analyzed the expression pattern of mRNAs or pre-mRNAs of all sarcomeric *Myh* (embryonic, neonatal, slow and adult fast) on isolated forelimbs myofibers at different stages. At E15.5, when myofibers were already innervated, embryonic primary myofibers expressed *Myh3* and *Myh8* mRNA (Supplementary Fig. 9a). At E18.5, secondary fetal myofibers (small fibers) displayed a higher accumulation of *Myh3* than of *Myh8. Myh3* mRNAs accumulated strongly in MTJ regions of myofibers at all stages, (Supplementary Fig. 9a, b) and after birth also at the center of myofibers, where NMJs are suspected to be localized (Fig. 7a and Supplementary Fig. 9a). These results suggested the specific transient activation of *Myh3* in NMJ and MTJ myonuclei, or its transient activation in newly accreted myonuclei in these regions of growing myofibers as proposed previously^38^. In contrast, *Myh8* mRNA was not regionalized along the myofiber at any stage. Adult f*Myh* pre-mRNAs were first detected in a few nuclei at E15.5 and their number increased drastically after E18.5 (Fig. 7b, c). At these different stages, most of the myonuclei expressed only one adult f*Myh* gene (Fig. 7d) without obvious regionalization along the myofiber. We observed mono-allelic expression before E18.5, and bi-allelic expression after P2 (higher transcription rates) (Fig. 7e). At P2 and P5, more than 90% of the nuclei were coordinated along the myofibers and expressed one f*Myh* gene (Fig. 7f). This percentage was similar to that of adult EDL, suggesting that f*Myh* gene coordination is established early when the expression is initiated. *Myh2* and *Myh4* mRNAs accumulated strongly from P5 but some areas of the fiber did not present mRNAs accumulation (Supplementary Fig. 10a). Slow *Myh7* mRNAs accumulated in all primary fibers at the MTJ level at E15.5 (Fig. 7g). After E18.5, the expression of *Myh7* was restricted to slow fibers, which homogeneously accumulated this mRNA specifically (Supplementary Fig. 10b). These experiments showed that the majority of myonuclei started to express adult f*Myh* genes at P2, a single gene being activated by nucleus, and that myonuclei in the growing myofiber were coordinated from their onset.

## Discussion

**Fig. 8:**
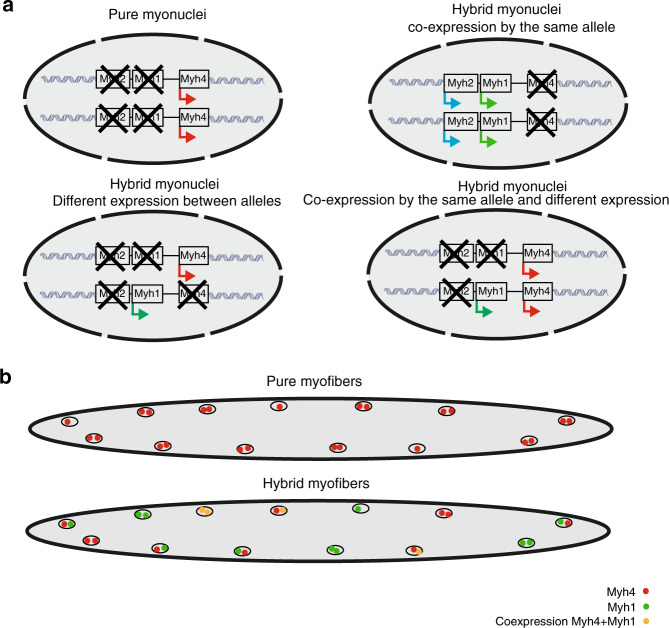
Diagram of *Myh* expression in pure and hybrid myonuclei and myofiber. **a** Diagram showing the different possibilities of *Myh* isoforms expression in a myonucleus from the results of our experiments. **b** Diagram showing the different possibilities of *Myh* isoforms expression in a myofiber from the results of our experiments.

By combining single-nucleus RNA-seq/ATAC-seq and FISH experiments, we established an atlas of the gene expression pattern and chromatin landscape of the different cell populations of adult skeletal muscles, including mononucleated cells and multinucleated myofibers. From our experiments, we estimated that myonuclei constitute ~68% of the nuclei present in adult skeletal muscles of 2 months old mice at rest. Recent single-cell RNA-seq studies of skeletal muscle^39,40^ have characterized the gene expression pattern of mononucleated cells of adult skeletal muscle, but failed to identify the transcription signature of multinucleated myofibers. The composition and gene expression of cell types identified by these reports is in agreement with our data, providing the same global signature of skeletal muscle at rest, identifying distinct populations of FAPs, tenocytes, immune cells, pericytes, adipocytes, smooth muscle cells, and myogenic stem cells. Expanding these studies, our results showed that myofibers are composed of three different types of myonuclei localized differently and expressing distinct sets of genes (Fig. 2), namely the MTJ, NMJ, and body myonuclei. The full transcriptome of these nuclei was to this day unknown, and we characterized numerous genes expressed specifically in these specialized myonuclei. We also characterized an unidentified population of *Titin* + myonuclei expressing *Runx1, Dysf*, *Ehd4*, *Flnc*, and *Myh9*. As Filamin C is involved in early stages of myofibrillar remodeling and repair, this myonuclei population, which had never been described could correspond to myonuclei in area of myofiber damage to support fast repair^41,42^. Our results demonstrated that most myonuclei express a single f*Myh* gene, and that in the majority of myofibers myonuclei coordinately express a specific set of genes, including this *Myh* gene (Fig. 8). Our RNAscope experiments also identified a feature of transcriptional control in myonuclei of adult myofibers. In fast skeletal muscles, the transcriptional activity of nuclei within each myofiber is finely coordinated. We showed that this coordination is dependent upon innervation and is established early during development.

Several reports have shown that apparently homogenous populations of cells present an important variability of gene expression. This variability can be the consequence of stochastic gene expression, different stages of cell cycle, or distinct subcategories of cells in the studied population^43^. In syncytia like osteoclasts and syncytiotrophoblasts, gene expression can be heterogeneous in nuclei that share the same cytoplasm^10,11^. We found that the transcription of adult f*Myh* genes in myonuclei along given myofibers was finely controlled during development, allowing the coordinated expression in their nuclei of a single f*Myh* gene. This transcriptional coordination could be a consequence of various processes. In *Drosophila*, one founder myonucleus was shown to reprogram newly fused myonuclei, inducing coordination of expression between nuclei of the same fiber^44^. Alternatively, distinct populations of preprogrammed myogenic stem cells with distinct potential^45–49^ could fuse (homotypic fusion^50^), activating a single *Myh* gene along the myofiber. Moreover, extracellular signals from surrounding cells may also intervene^51^. The de-coordination of myonuclei after denervation observed in our study supports the importance of innervation in the coordination process during development. A better understanding of the matching between motoneuron subtypes and myofiber subtypes that operates during development^52^ may help understand how neuromuscular specialization contributes to the onset of coordination.

Adult myofibers are plastic and can adapt their contractile and metabolic properties depending on external stimuli by activating or repressing a set of specific genes, such as the *Myh* genes^14,15^. These changes occur in a sequential order recapitulating the organization of the genes at the fast *Myh* locus (*Myh4-Myh1-Myh2*)^15^. In this report, we showed that most myonuclei expressed only one *Myh* isoform at a given time (Fig. 4). This robust and exclusive expression is reminiscent of the control of the *ß-Globin* genes by a locus control region^23^. The regulation of the f*Myh* locus is not fully understood and could be controlled like the *ß*-*Globin* genes by a locus control region, such as a super enhancer^53,54^, activating the expression of associated embryonic, fetal and adult *Globin* genes in a temporal order. Transient transfection of adult mouse TA and soleus with *GFP* reporters under the control of 1 kb upstream of each *Myh2*, *Myh1* and *Myh4* promoters recapitulate their specific expression in the corresponding MYH2, MYH1 and MYH4 myofibers, respectively^55,56^. This suggests that each f*Myh* promoter possesses its own fiber specific cis-regulatory elements. Accordingly, in *Myh4* + nuclei, we observed in snATAC-seq experiments a higher chromatin opening in *Myh4* than in *Myh1* and *Myh2* genes. In *Myh4* + nuclei, we failed to detect chromatin opening at the *Myh1* and *Myh2* promoter regions, suggesting the absence of TF binding. In soleus myofibers with a high proportion of *Myh1* and *Myh2* co-expression (where *Myh1* and *Myh2* pre-mRNAs are detected in many myonuclei), we mainly observed *Myh1* and *Myh2* pre-mRNAs as two dots in the myonuclei, suggesting that each allele transcribed a single gene, and that these two adjacent f*Myh* genes could not be transcribed simultaneously. Whether this exclusive expression reflects the competition for a shared enhancer at the locus, as already illustrated for the *ß-Globin* genes^23^, is an interesting possibility.

The coordinated expression of a single isoform of *Myh* could be due to a similar combination of transcription activators and repressors concentration in these myonuclei, affecting all the genes that they express, or only those with high expression, excluding stochastic expression as previously proposed^9^. In support of this, snRNA-seq experiments showed that specific genes are co-expressed with *Myh4*, such as genes coding for other sarcomeric proteins, proteins associated with metabolism or associated with calcium handling. The snRNA-seq results showed harmonious non-random gene expression pattern in each nucleus of the myofiber, with some variability between muscles with distinct subprograms among *Myh4* + myonuclei. In the mouse, denervation induces muscle atrophy, associated with a fast to slow transition of *Myh* at the protein level^32^. These changes occur also at the pre-mRNA level, as shown in the present study. We observed that transitions after denervation were sequential, *Myh4* + fibers expressing *Myh1* but not *Myh2*, and some nuclei switching their *Myh* expression profile earlier than others. Thirty days after denervation, myofibers remained uncoordinated showing that the increased number of hybrid fibers observed in EDL 7 days after denervation was not due to a transitional state but that innervation was required to maintain the coordination of f*Myh* expression. The atrophy of myofibers induced by denervation involves the activation of protein degradation pathways and the repression of protein synthesis^57^. We observed an increased number of f*Myh*-negative nuclei as well as a decreased bi-allelic expression of f*Myh* after muscle denervation, suggesting that downregulation of f*Myh* expression may participate as well in this atrophy.

The loss of coordination in myonuclei of adult myofibers after denervation observed in the present study is consistent with the role of motoneurons in controlling adult muscle physiology^14,15,30,32,58,59^. Calcium is a diffusible signal distributed throughout myofibers and a potential candidate for the control of specific muscle gene coordination. Resting Ca^2+^ concentration differs in fast and slow fibers and is modulated by slow or fast motoneuron firing^60–62^. Ca^2+^ transients following slow motoneuron stimulation activate specific signaling pathways^14,63^ among which Calcineurin phosphatase that controls the nucleo-cytoplasmic localization of NFATc1 in slow myofibers important for the expression of slow muscle genes^64,65^. MYOD activity as well is modulated by motoneuron activity and phosphorylation of its T115 by slow motoneuron activation impairs its DNA binding activity in the soleus^66^. In contrast, fast-twitch motoneurons, characterized by phasic high-frequency firing do not activate Calcineurin phosphatase, suggesting that other signaling pathways relay motoneurons firing information in fast myofibers. Furthermore, fast-subtypes myofibers are innervated by fast-subtypes motoneurons, including fast fatigable (FF, innervating MYH4 myofibers), fast intermediate (FI, innervating MYH1 myofibers) and fast resistant (FR, innervating MYH2 myofibers)^67–69^. How these distinct classes of fast motoneurons control the fast-muscle subtypes phenotype and myonuclei coordination is presently unknown^32,59,67,70^. Whether Ca^2+^ concentrations are similar along the myofiber and how denervation may perturb this basal intracellular Ca^2+^ concentrations are potential important issues to understand myonuclei coordination. Several proteins controlling intracellular Ca^2+^ concentration in myofibers (Calsequestrin 1 et 2, Serca1 and 2, Parvalbumin, Calmodulin, Phospholamban, Myozenin 1 and 2, Sarcalumenin, IP3R) are differentially expressed in fast and slow myofibers, and induce a variety of local Ca^2+^ concentration gradients that may modulate the activity of different transcription factors^71–74^. Here, we showed that the transcription factor SIX1 accumulates in all myonuclei of EDL myofibers and participates in the coordination of f*Myh* expression. The mechanisms controlling SIX1 nuclear accumulation specifically in fast myofibers remain to be established. We observed by snRNA-seq an increased expression of the SIX cofactor EYA4 in *Myh4* + myonuclei. Whether the SIX1-EYA4 transcription complex activity may be controlled by motoneuron firing frequency and participates in myonuclei coordination is an interesting possibility that may explain its role in the control of the adult fast phenotype^75^.

The genome-wide chromatin landscape of adult muscles had not been evaluated previously at the myofiber level, due to the lack of adapted experimental protocols^26^. We show here that snATAC-seq is a powerful tool to overcome this limitation and that it allows to detect all the cell populations identified by snRNA-seq in adult skeletal muscles, including myofibers. We identified chromatin accessibility domains coherent with the defined patterns of gene expression, specific of fast-subtypes or slow myonuclei, of MTJ or body myonuclei, as well as of other cell types in skeletal muscle. This analysis also allowed to identify enriched TF-binding sites in ATAC-peaks specific for each myonuclei population. We identified TF-binding motifs known to participate in muscle genes regulation in slow and fast myofibers, among which the NFATc^65^, MEF2^76^ and SIX^35,77,78^ families, and nuclear receptors of the ERR-ß/γ^79^ and the NR4A1^80^ families. The differential TF ATAC-peaks enrichment observed between fast-subtypes myonuclei sheds some light on the basis of their specialization. We identified TF DNA binding motifs, which had never been implicated in muscle genes regulation, such as those recognized by the b-ZIP family of NRL/MAF transcription factors^81^. Accordingly, we identified *c-Maf* mRNAs enriched in the *Myh4* + myonuclei in our snRNA-seq, suggesting that this TF may contribute to the fine-tuning of muscle gene expression in specialized fast myonuclei, in synergy with SIX homeoproteins. Serria et al showed earlier that *c-Maf* expression is upregulated in myogenic cells during their differentiation in culture, but the role of *c-Maf* in adult muscle physiology is presently unknown^82^.

Last, in addition to defining the exact transcriptional landscape within skeletal muscle, the snRNA-seq and snATAC-seq methods presented here open new perspectives to characterize in the future the crosstalk between myofibers and their environment, such as with associated myogenic stem cells in pathophysiological conditions, to date poorly investigated due to technical limits.

## Methods

### Animals

Animal experimentations were carried out in strict accordance with the European STE 123 and the French national charter on the Ethics of Animal Experimentation. Protocols were approved by the Ethical Committee of Animal Experiments of the Institut Cochin, CNRS UMR 8104, INSERM U1016, and by the Ministère de l′éducation nationale, de l′enseignement et de la recherche, no. APAFIS#15699-2018021516569195. We used 6- to 8- week-old C57black6N mouse female for most of our experiments. Mice were anesthetized with intraperitoneal injections of ketamine and xylazine and with subcutaneous buprecare injections before denervation was performed by sectioning of the sciatic nerve in one leg.

### FACS sorting of nuclei from adult skeletal muscle for single-nucleus RNA-seq

For the mix condition 6 tibialis, 6 gastrocnemius, 6 soleus, 6 plantaris, and 6 EDL were pulled together. For the soleus condition, 24 soleus, for the tibialis condition 6 tibialis and for the quadriceps condition 6 quadriceps were pulled together. Muscles were dissected and pulled in cold phosphate-buffered saline (PBS) with 0.2 U/μl RNase inhibitor. PBS was removed and muscles were minced in 1 ml cold lysis buffer (10 mM Tris-HCl pH7.5, 10 mM NaCl, 3 mM MgCl_2_, and 0.1% NonidetTM P40 in Nuclease-Free Water) with scissors. After 2 min, 4 ml of cold lysis buffer were added and muscles were lysed for 3 min at +4 °C. Nine milliliter of cold wash buffer (PBS, BSA 2% and 0.2 U/μl RNase inhibitor from Roche) was added and the lysate was dounced with 10 strokes of loose pestle avoiding too much pressure and air bubbles. The homogenate was filtered with 70 and 40 μm cell strainers. Nuclei were pelleted by centrifugation for 5 min at 500 × *g* at +4 °C. Nuclei were washed to remove ambient RNAs in 1 ml of cold wash buffer, transferred in a 1.5 ml Eppendorf tube and centrifuged 5 min at 500 × *g* at +4 °C. The pellet was resuspended in 250 μl of wash buffer and stained during 15 min in the dark at +4 °C with DAPI (10 μg/ml). Then the nuclei were washed with 1 ml of wash buffer, centrifuged 5 min at 500 × *g* at +4 °C, resuspended in 300 μl of wash buffer and filtered with 30 μm cell strainers. Nuclei were then FACS sorted to exclude debris with a BD FACSAria III and the BD FACSDIVA software. More than 200,000 nuclei were collected in a tube containing 200 μl of wash buffer.

### Single-nucleus RNA-seq from skeletal muscle

After FACS sorting, nuclei were centrifuged 5 min at 500 × *g* at +4 °C, resuspended in 15 μl of wash buffer and counted with a hemocytometer. The concentration of nuclei was then adjusted to 1000 nuclei/μl with wash buffer. We loaded around 4000 nuclei per condition into the 10x Chromium Chip. We used the Single-Cell 3′ Reagent Kit v3 according to the manufacturer’s protocol. GEM-Reverse Transcription (GEM-RT) was performed in a thermal cycler: 53 °C for 45 min, 85 °C for 5 min. Post GEM-RT Cleanup using DynaBeads MyOne Silane Beads was followed by cDNA amplification (98 °C for 3 min, cycled 12 × 98 °C for 15 s, 67 °C for 20 s, 72 °C for 1 min). After a cleanup with SPRIselect Reagent Kit and fragment size estimation with High-SensitivityTM HS DNA kit runned on 2100 Bioanalyzer (Agilent), the libraries were constructed by performing the following steps: fragmentation, end-repair, A-tailing, SPRIselect cleanup, adaptor ligation, SPRIselect cleanup, sample index PCR, and SPRIselect size selection. The fragment size estimation of the resulting libraries was assessed with High-SensitivityTM HS DNA kit runned on 2100 Bioanalyzer (Agilent) and quantified using the QubitTM double stranded DNA (dsDNA) High-Sensitivity HS assay (ThermoFisher Scientific). Libraries were then sequenced by pair with a HighOutput flowcel using an Illumina Nextseq 500 with the following mode: 26base-pairs (bp) (10x Index + UMI), 8 bp (i7 Index), and 57 bp (Read 2).

### Single-nucleus RNA-seq analysis

A minimum of 50,000 reads per nucleus were sequenced and analyzed with Cell Ranger Single-Cell Software Suite 3.0.2 by 10x Genomics. Raw base call files from the Nextseq 500 were demultiplexed with the cellranger mkfastq pipeline into library-specific FASTQ files. The FASTQ files for each library were then processed independently with the cellranger count pipeline with parameter set to default. The single-nucleus RNA-seq assay captures unspliced pre-mRNA, as well as mature mRNA. To include these intronic reads in our analysis, we created a custom “pre-mRNA” reference package, listing each gene transcript locus as an exon with cellranger mkgtf and cellranger mkref according to 10x genomics instruction (https://support.10xgenomics.com/single-cell-gene-expression/software/pipelines/latest/advanced/references). We used a reference genome built against mouse mm10, Sequence: GRCm38 Ensembl 93. We used STAR21 to align cDNA reads against our reference genome. Once aligned, barcodes associated with these reads—cell identifiers and Unique Molecular Identifiers (UMIs), underwent filtering and correction. Reads associated with retained barcodes were quantified and used to build a transcript count table. The subsequent visualizations, clustering and differential expression tests were performed in R (v 3.4.3) using Seurat (v3.0.2)^25^. Quality control on aligned and counted reads was performed keeping cells with >200 and <2500 nFeature RNA and <5% mitochondrial genes. We get 6477 nuclei from the mix of tibialis, EDL, gastrocnemius, plantaris and soleus, 1335 nuclei from quadriceps, 4838 nuclei from tibialis, 2517 nuclei from soleus. We detected the expression of approximately one thousand genes per nucleus in each of these muscles (Supplementary Fig. 1).

### Single-nucleus ATAC-seq from skeletal muscle

We used the 10x genomic nuclei Isolation for Single-Cell ATAC Sequencing protocol (CG000169|Rev B) with the following modifications: 12 quadriceps and 12 soleus were dissected and pulled in cold PBS. PBS was removed and muscles were minced 2 min in 1 ml of cold ATAC-lysis buffer (10 mM Tris-HCl pH7.4, 10 mM NaCl, 3 mM MgCl_2_, 1% BSA and 0.1% Tween-20 in Nuclease-Free Water). Six milliliter of cold ATAC-lysis buffer were added and muscles were lysed on ice. After 3 min the lysate was dounced with 10 strokes of loose pestle avoiding too much pressure and air bubbles. After douncing, 8 ml of wash buffer were added and the homogenate was filtered with 70, 40, and 20 μm cell strainers. Nuclei were pelleted by centrifugation for 5 min at 500 × *g* at +4 °C. Next, we used the Chromium Single-Cell ATAC kit according to the manufacturer’s protocol. Nuclei were resuspended in nuclei buffer from the kit, transposed 1 h at 37 °C. We loaded around 6000 nuclei into the 10*X* Chromium Chip. GEM incubation and amplification were performed in a thermal cycler: 72 °C for 5 min, 98 °C for 30 s, and 12 repeated cycles of 98 °C for 10 s, 59 °C for 30 s, and 72 °C for 1 min. Post GEM Cleanup using DynaBeads MyOne Silane Beads was followed by library construction (98 °C for 45 s, cycled 12 × 98 °C for 20 s, 67 °C for 30 s, 72 °C for 1 min). The libraries were constructed by adding sample index PCR, and SPRIselect size selection. The fragment size estimation of the resulting libraries was assessed with High-SensitivityTM HS DNA kit runned on 2100 Bioanalyzer (Agilent) and quantified using the QubitTM dsDNA High-Sensitivity HS assay (ThermoFisher Scientific). Libraries were then sequenced by pair with a HighOutput flowcel using an Illumina Nextseq 500.

### Single-nucleus ATAC-seq analysis

A minimum of 10,000 reads per nucleus were sequenced and analyzed with Cell Ranger Single-Cell Software Suite 3.0.2 by 10x Genomics. Raw base call files from the Nextseq 500 were demultiplexed with the cellranger-atac mkfastq pipeline into library-specific FASTQ files. The FASTQ files for each library were then processed independently with the cellranger count pipeline. This pipeline used STAR21 to align reads to the Mus musculus genome. Once aligned, barcodes associated with these reads—cell identifiers and Unique Molecular Identifiers (UMIs), underwent filtering and correction. The subsequent visualizations, clustering and differential expression tests were performed in R (v 3.4.3) using Seurat (v3.0.2)^25^, Signac (v0.2.4) (https://github.com/timoast/signac) and Chromvar (v1.1.1)^27^. Quality control on aligned and counted reads was performed keeping cells with peak_region_fragments >3000 reads and <100,000, pct reads in peaks >15, blacklist ratio <0.025, nucleosome_signal <10, and TSS.enrichment >2. We get 6037 nuclei in total and we detected 132,966 peaks (Supplementary Fig. 1). The motif activity score was analyzed by running chromVAR (Fig. 3c–e). The script used for the Signac analysis is available here: https://github.com/matthieudossantos/Single-nuclei-RNAseq-and-single-nuclei-ATACseq-script-for-Seurat/blob/master/script.

### FISH with amplification (RNAscope) on isolated fibers

RNAscope® Multiplex Fluorescent Assay V2 was used to visualize f*Myh* pre-mRNAs and mRNAs, and *AldolaseA* and *Idh2* pre-mRNAs. Twenty different pairs of probes against the first intron of each f*Myh, AldolaseA* and *Idh2* pre-mRNAs were designed by ACDbio. Muscles were dissected and immediately fixed in 4% PFA at +4 °C for 30 min. After fixation muscles were washed three times in PBS for 5 min. Myofibers were dissociated mechanically with small tweezers and fixed onto Superfrost plus slides (Thermo Fischer) coated with Cell-Tak (Corning) by dehydration at +55 °C during 5 min. Slides were then proceeded according to the manufacturer’s protocol: ethanol dehydration, 10 min of H_2_O_2_ treatment and 30 min of protease IV treatment. After hybridization and revelation, the fibers were mounted under a glass coverslip with Prolong Gold Antifade Mountant (Thermofischer). Myofibers were imaged with a Leica DMI6000 confocal microscope composed by an Okogawa CSU-X1M1 spinning disk and a CoolSnap HQ2 Photometrics camera. Images were analyzed with Fiji Cell counter program.

### Immunohistochemistry

For immunostaining against MYH4, MYH2, MYH7, and Laminin, adult legs without fixation and without skin were embedded with TissuTEK OCT, and directly frozen in cold isopentane cooled in liquid nitrogen. Muscles were conserved at −80 °C and cut with Leica cryostat 3050s with a thickness of 10 μm. Cryostat sections were washed three times 5 min with PBS and then incubated with blocking solution (PBS and 10% goat serum) 30 min at room temperature. Sections were incubated overnight with primary antibody solution at +4 °C, then washed three times for 5 min with PBS and incubated with secondary antibody solution 1 h at room temperature. Sections were further washed three times for 5 min and mounted with Mowiol solution and a glass coverslip. Images were collected with an Olympus BX63F microscope and a Hamamatsu ORCA-Flash 4.0 camera. Images were analyzed with ImageJ program. The references of the antibodies used are listed in Supplementary Table 1.

### Statistical analysis

The graphs represent mean values ± SEM. Significant differences between mean values were evaluated using two-way ANOVA with Graphpad 6 software and student *t* test for Fig. 6g.

### Reporting summary

Further information on research design is available in the Nature Research Reporting Summary linked to this article.

## Supplementary information

Supplementary Information

Supplementary Data 1

Description of Additional Supplementary Files

Reporting Summary

## Data Availability

The authors declare that all data supporting the findings of this study are available within the article and its supplementary information files or from the corresponding author upon reasonable request. All single nucleus RNA-seq and Single-nucleus ATAC-seq data are available in the NCBI Gene Expression Omnibus (GEO) database under accession GSE150065. Source data are available.

## Source data

Source Data

## Code availability

The script used for the Seurat analysis is available here: [https://github.com/matthieudossantos/Single-nuclei-RNAseq-and-single-nuclei-ATACseq-script-for-Seurat/blob/master/script_single_nuclei_RNAseq.r].

## References

[1] Bruusgaard JC, Liestol K, Ekmark M, Kollstad K, Gundersen K (2003). Number and spatial distribution of nuclei in the muscle fibres of normal mice studied in vivo. J. Physiol..

[2] Kim N, Burden SJ (2008). MuSK controls where motor axons grow and form synapses. Nat. Neurosci..

[3] Fontaine B, Sassoon D, Buckingham M, Changeux JP (1988). Detection of the nicotinic acetylcholine receptor alpha-subunit mRNA by in situ hybridization at neuromuscular junctions of 15-day-old chick striated muscles. EMBO J..

[4] Thomas JL (2015). PAK1 and CtBP1 regulate the coupling of neuronal activity to muscle chromatin and gene expression. Mol. Cell Biol..

[5] Jacobson C, Cote PD, Rossi SG, Rotundo RL, Carbonetto S (2001). The dystroglycan complex is necessary for stabilization of acetylcholine receptor clusters at neuromuscular junctions and formation of the synaptic basement membrane. J. Cell Biol..

[6] Koch M (2004). A novel marker of tissue junctions, collagen XXII. J. Biol. Chem..

[7] Charvet B (2013). Knockdown of col22a1 gene in zebrafish induces a muscular dystrophy by disruption of the myotendinous junction. Development.

[8] Cutler, A. A., Jackson, J. B., Corbett, A. H. & Pavlath, G. K. Non-equivalence of nuclear import among nuclei in multinucleated skeletal muscle cells. *J. Cell Sci.* **131**, 10.1242/jcs.207670 (2018). 10.1242/jcs.207670 PMC5826044 29361530

[9] Newlands S (1998). Transcription occurs in pulses in muscle fibers. Genes Dev..

[10] Youn MY, Takada I, Imai Y, Yasuda H, Kato S (2010). Transcriptionally active nuclei are selective in mature multinucleated osteoclasts. Genes Cells.

[11] Ellery PM, Cindrova-Davies T, Jauniaux E, Ferguson-Smith AC, Burton GJ (2009). Evidence for transcriptional activity in the syncytiotrophoblast of the human placenta. Placenta.

[12] Braun T, Gautel M (2011). Transcriptional mechanisms regulating skeletal muscle differentiation, growth and homeostasis. Nat. Rev. Mol. Cell Biol..

[13] Greising SM, Gransee HM, Mantilla CB, Sieck GC (2012). Systems biology of skeletal muscle: fiber type as an organizing principle. Wiley Interdiscip. Rev. Syst. Biol. Med..

[14] Gundersen K (2011). Excitation-transcription coupling in skeletal muscle: the molecular pathways of exercise. Biol. Rev. Camb. Philos. Soc..

[15] Schiaffino S, Reggiani C (2011). Fiber types in mammalian skeletal muscles. Physiol. Rev..

[16] Shrager JB (2000). Human skeletal myosin heavy chain genes are tightly linked in the order embryonic-IIa-IId/x-ILb-perinatal-extraocular. J. Muscle Res. Cell Motil..

[17] LaFramboise WA (1991). Emergence of the mature myosin phenotype in the rat diaphragm muscle. Dev. Biol..

[18] Murgia M (2017). Single muscle fiber proteomics reveals fiber-type-specific features of human muscle aging. Cell Rep..

[19] Bottinelli R, Betto R, Schiaffino S, Reggiani C (1994). Maximum shortening velocity and coexistence of myosin heavy chain isoforms in single skinned fast fibres of rat skeletal muscle. J. Muscle Res. Cell Motil..

[20] Medler, S. Mixing it up: the biological significance of hybrid skeletal muscle fibers. *J. Exp. Biol.* **222**, 10.1242/jeb.200832 (2019). 10.1242/jeb.200832 31784473

[21] Rinaldi C (2008). Intergenic bidirectional promoter and cooperative regulation of the IIx and IIb MHC genes in fast skeletal muscle. Am. J. Physiol. Regul. Integr. Comp. Physiol..

[22] van Rooij E (2009). A family of microRNAs encoded by myosin genes governs myosin expression and muscle performance. Dev. Cell.

[23] Palstra RJ, de Laat W, Grosveld F (2008). Beta-globin regulation and long-range interactions. Adv. Genet.

[24] Fukaya T, Lim B, Levine M (2016). Enhancer control of transcriptional bursting. Cell.

[25] Stuart T (2019). Comprehensive integration of single-*cell data*. Cell.

[26] Ramachandran K (2019). Dynamic enhancers control skeletal muscle identity and reprogramming. PLoS Biol..

[27] Schep AN, Wu B, Buenrostro JD, Greenleaf WJ (2017). chromVAR: inferring transcription-factor-associated accessibility from single-cell epigenomic data. Nat. Methods.

[28] Salminen M, Lopez S, Maire P, Kahn A, Daegelen D (1996). Fast-muscle-specific DNA-protein interactions occurring in vivo at the human aldolase A M promoter are necessary for correct promoter activity in transgenic mice. Mol. Cell Biol..

[29] Murgia M (2015). Single muscle fiber proteomics reveals unexpected mitochondrial specialization. EMBO Rep..

[30] Rowan SL (2012). Denervation causes fiber atrophy and myosin heavy chain co-expression in senescent skeletal muscle. PLoS ONE.

[31] Dix DJ, Eisenberg BR (1990). Myosin mRNA accumulation and myofibrillogenesis at the myotendinous junction of stretched muscle fibers. J. Cell Biol..

[32] Agbulut O (2009). Slow myosin heavy chain expression in the absence of muscle activity. Am. J. Physiol. Cell Physiol..

[33] Lang F (2018). Single muscle fiber proteomics reveals distinct protein changes in slow and fast fibers during muscle atrophy. J. Proteome Res..

[34] Raffaello A (2006). Denervation in murine fast-twitch muscle: short-term physiological changes and temporal expression profiling. Physiol. Genomics.

[35] Sakakibara I, Santolini M, Ferry A, Hakim V, Maire P (2014). Six homeoproteins and a Iinc-RNA at the fast MYH locus lock fast myofiber terminal phenotype. PLoS Genet..

[36] Sakakibara I (2016). Six1 homeoprotein drives myofiber type IIA specialization in soleus muscle. Skelet. Muscle.

[37] Agbulut O, Noirez P, Beaumont F, Butler-Browne G (2003). Myosin heavy chain isoforms in postnatal muscle development of mice. Biol. Cell.

[38] Zhang M, McLennan IS (1995). During secondary myotube formation, primary myotubes preferentially absorb new nuclei at their ends. Dev. Dyn..

[39] Giordani L (2019). High-dimensional single-cell cartography reveals novel skeletal muscle-resident cell populations. Mol. Cell.

[40] Dell’Orso, S. et al. Single cell analysis of adult mouse skeletal muscle stem cells in homeostatic and regenerative conditions. *Development* **146**, 10.1242/dev.174177 (2019). 10.1242/dev.174177 PMC6602351 30890574

[41] Barthelemy F, Defour A, Levy N, Krahn M, Bartoli M (2018). Muscle cells fix breaches by orchestrating a membrane repair ballet. J. Neuromuscul. Dis..

[42] Leber Y (2016). Filamin C is a highly dynamic protein associated with fast repair of myofibrillar microdamage. Hum. Mol. Genet..

[43] Martinez-Jimenez CP (2017). Aging increases cell-to-cell transcriptional variability upon immune stimulation. Science.

[44] Bataille L, Boukhatmi H, Frendo JL, Vincent A (2017). Dynamics of transcriptional (re)-programming of syncytial nuclei in developing muscles. BMC Biol..

[45] Rosenblatt JD, Parry DJ, Partridge TA (1996). Phenotype of adult mouse muscle myoblasts reflects their fiber type of origin. Differentiation.

[46] Kalhovde JM (2005). “Fast” and “slow” muscle fibres in hindlimb muscles of adult rats regenerate from intrinsically different satellite cells. J. Physiol..

[47] Lee KY (2015). Tbx15 controls skeletal muscle fibre-type determination and muscle metabolism. Nat. Commun..

[48] Wang JH (2015). Heterogeneous activation of a slow myosin gene in proliferating myoblasts and differentiated single myofibers. Dev. Biol..

[49] Ngo-Muller V, Bertrand A, Concordet JP, Daegelen D (2003). Mouse muscle identity: the position-dependent and fast fiber-specific expression of a transgene in limb muscles is methylation-independent and cell-autonomous. Dev. Dyn..

[50] Pin CL, Hrycyshyn AW, Rogers KA, Rushlow WJ, Merrifield PA (2002). Embryonic and fetal rat myoblasts form different muscle fiber types in an ectopic in vivo environment. Dev. Dyn..

[51] Anakwe K (2003). Wnt signalling regulates myogenic differentiation in the developing avian wing. Development.

[52] Rafuse V, Landmesser L (2000). The pattern of avian intramuscular nerve branching is determined by the innervating motoneuron and its level of polysialic acid. J. Neurosci..

[53] Whyte WA (2013). Master transcription factors and mediator establish super-enhancers at key cell identity genes. Cell.

[54] Hnisz D, Shrinivas K, Young RA, Chakraborty AK, Sharp PA (2017). A phase separation model for transcriptional control. Cell.

[55] Allen DL, Sartorius CA, Sycuro LK, Leinwand LA (2001). Different pathways regulate expression of the skeletal myosin heavy chain genes. J. Biol. Chem..

[56] Allen DL, Weber JN, Sycuro LK, Leinwand LA (2005). Myocyte enhancer factor-2 and serum response factor binding elements regulate fast Myosin heavy chain transcription in vivo. J. Biol. Chem..

[57] Schiaffino S, Dyar KA, Ciciliot S, Blaauw B, Sandri M (2013). Mechanisms regulating skeletal muscle growth and atrophy. FEBS J..

[58] Hennig R, Lomo T (1985). Firing patterns of motor units in normal rats. Nature.

[59] Salviati G, Biasia E, Aloisi M (1986). Synthesis of fast myosin induced by fast ectopic innervation of rat soleus muscle is restricted to the ectopic endplate region. Nature.

[60] Eccles JC, Eccles RM, Lundberg A (1957). Durations of after-hyperpolarization of motoneurones supplying fast and slow muscles. Nature.

[61] Olson EN, Williams RS (2000). Remodeling muscles with calcineurin. Bioessays.

[62] Eusebi F, Miledi R, Takahashi T (1980). Calcium transients in mammalian muscles. Nature.

[63] Wu H (2002). Regulation of mitochondrial biogenesis in skeletal muscle by CaMK. Science.

[64] Chin ER (1998). A calcineurin-dependent transcriptional pathway controls skeletal muscle fiber type. Genes Dev..

[65] Tothova J (2006). NFATc1 nucleocytoplasmic shuttling is controlled by nerve activity in skeletal muscle. J. Cell Sci..

[66] Ekmark M, Rana ZA, Stewart G, Hardie DG, Gundersen K (2007). De-phosphorylation of MyoD is linking nerve-evoked activity to fast myosin heavy chain expression in rodent adult skeletal muscle. J. Physiol..

[67] Kanning KC, Kaplan A, Henderson CE (2010). Motor neuron diversity in development and disease. Annu. Rev. Neurosci..

[68] Muller D (2014). Dlk1 promotes a fast motor neuron biophysical signature required for peak force execution. Science.

[69] Fogarty MJ, Gonzalez Porras MA, Mantilla CB, Sieck GC (2019). Diaphragm neuromuscular transmission failure in aged rats. J. Neurophysiol..

[70] Pun S, Santos AF, Saxena S, Xu L, Caroni P (2006). Selective vulnerability and pruning of phasic motoneuron axons in motoneuron disease alleviated by CNTF. Nat. Neurosci..

[71] Frey N (2008). Calsarcin-2 deficiency increases exercise capacity in mice through calcineurin/NFAT activation. J. Clin. Invest..

[72] Berchtold MW, Brinkmeier H, Muntener M (2000). Calcium ion in skeletal muscle: its crucial role for muscle function, plasticity, and disease. Physiol. Rev..

[73] Casas M (2010). IP(3)-dependent, post-tetanic calcium transients induced by electrostimulation of adult skeletal muscle fibers. J. Gen. Physiol..

[74] Racay P, Gregory P, Schwaller B (2006). Parvalbumin deficiency in fast-twitch muscles leads to increased ‘slow-twitch type’ mitochondria, but does not affect the expression of fiber specific proteins. FEBS J..

[75] Maire P (2020). Myogenesis control by SIX transcriptional complexes. Semin. Cell Dev. Biol..

[76] Potthoff MJ, Olson EN (2007). MEF2: a central regulator of diverse developmental programs. Development.

[77] Grifone R (2004). Six1 and Eya1 expression can reprogram adult muscle from the slow-twitch phenotype into the fast-twitch phenotype. Mol. Cell Biol..

[78] Santolini M (2016). MyoD reprogramming requires Six1 and Six4 homeoproteins: genome-wide cis-regulatory module analysis. Nucleic Acids Res..

[79] Rangwala SM (2010). Estrogen-related receptor gamma is a key regulator of muscle mitochondrial activity and oxidative capacity. J. Biol. Chem..

[80] Chao LC (2007). Nur77 coordinately regulates expression of genes linked to glucose metabolism in skeletal muscle. Mol. Endocrinol..

[81] Katsuoka F, Yamamoto M (2016). Small Maf proteins (MafF, MafG, MafK): history, structure and function. Gene.

[82] Serria MS (2003). Regulation and differential expression of the c-maf gene in differentiating cultured cells. Biochem. Biophys. Res. Commun..

