# Supplementary material for: Single-nucleus RNA-seq and FISH identify coordinated transcriptional activity in mammalian myofibers
Source: Nat Commun. 2020 Oct 9;11:5102. doi: 10.1038/s41467-020-18789-8 (PMC7547110; doi:10.1038/s41467-020-18789-8)
Supplement: Supplementary file 1 — Supplementary Information [file 41467_2020_18789_MOESM1_ESM.pdf]

Supplementary Information

Single-nucleus RNA-seq and FISH identify coordinated transcriptional activity in mammalian myofibers.

Matthieu Dos Santos et al.

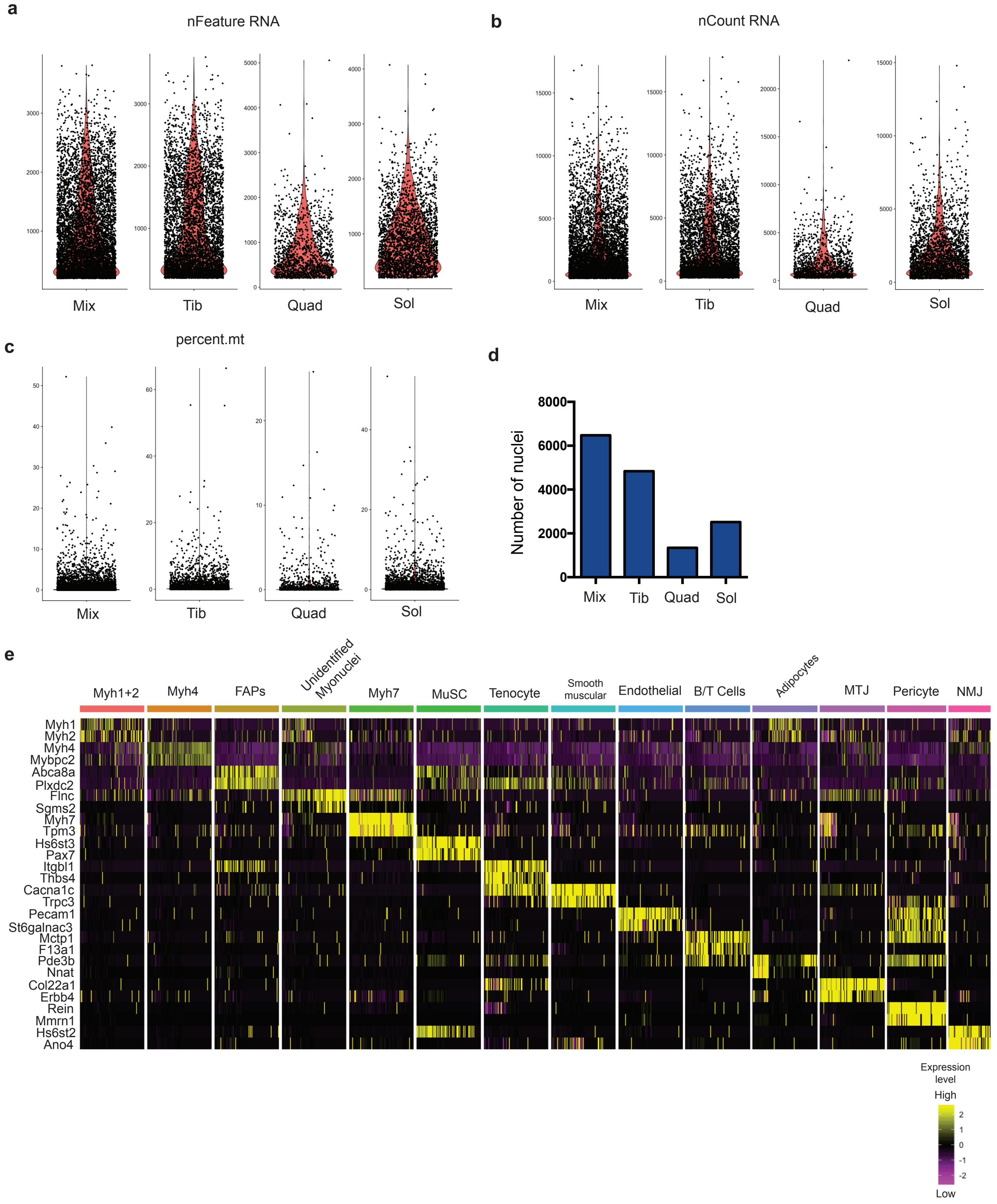

Supplementary Figure 1. Quality controls of the snRNA-seq experiments from Fig. 1. a Number of detected genes per sample using exonic and intronic sequences. **b** Number of reads per sample using exonic and intronic sequences. **c** Percentage of mitochondrial genes detected per sample. **d** Number of nuclei per sample after selecting nuclei that have unique feature counts between 2,500 and 200 and less than 5% mitochondrial counts. For each panel: Mix: mix of tibialis, EDL, gastrocnemius, plantaris and soleus; Tib: Tibialis; Quad: Quadriceps; Sol: Soleus. **e** Heatmap of the top two genes preferentially expressed (yellow) in each population of nuclei. The values correspond to z-scores of normalized counts.

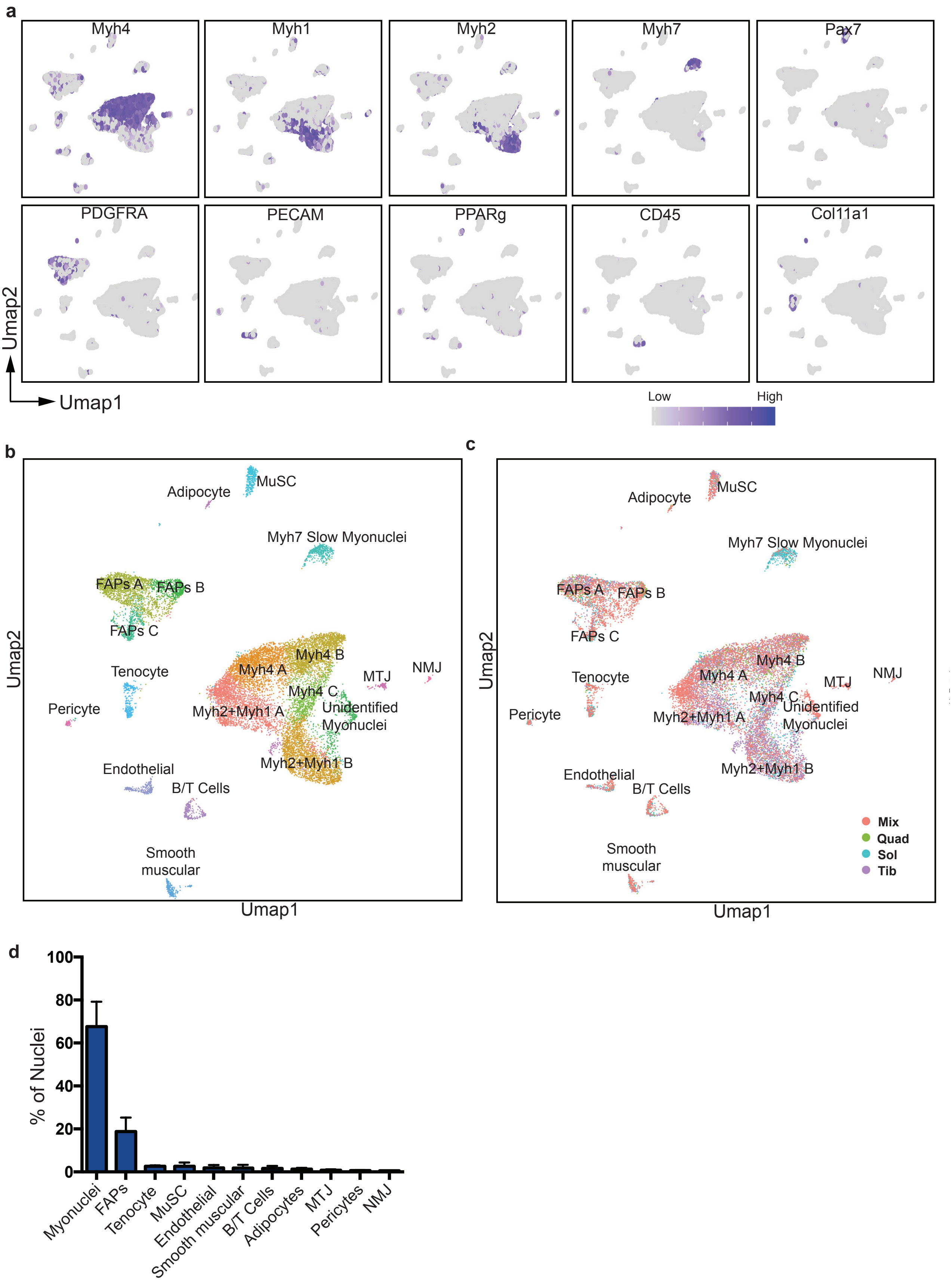

Supplementary Figure 2. Origin and identity of the nuclei populations defined in Fig. 1. a Same Umap diagram than in Fig. 1b, showing the expression of several markers used to identify the different cell types populations. The intensity of the blue color depends on the level of expression of the gene. **b** Same Umap diagram than in Fig. 1b showing the total number of cluster detected. **c** Same Umap diagram than in Fig. 1b showing the origin of each nucleus. **d** Quantification of the percentage of cell types found by snRNA-seq in the distinct studied muscles. MuSC: Skeletal muscle stem cells; FAPs: Fibro-adipogenic progenitors; MTJ: myotendinous junction; NMJ: neuromuscular junction.

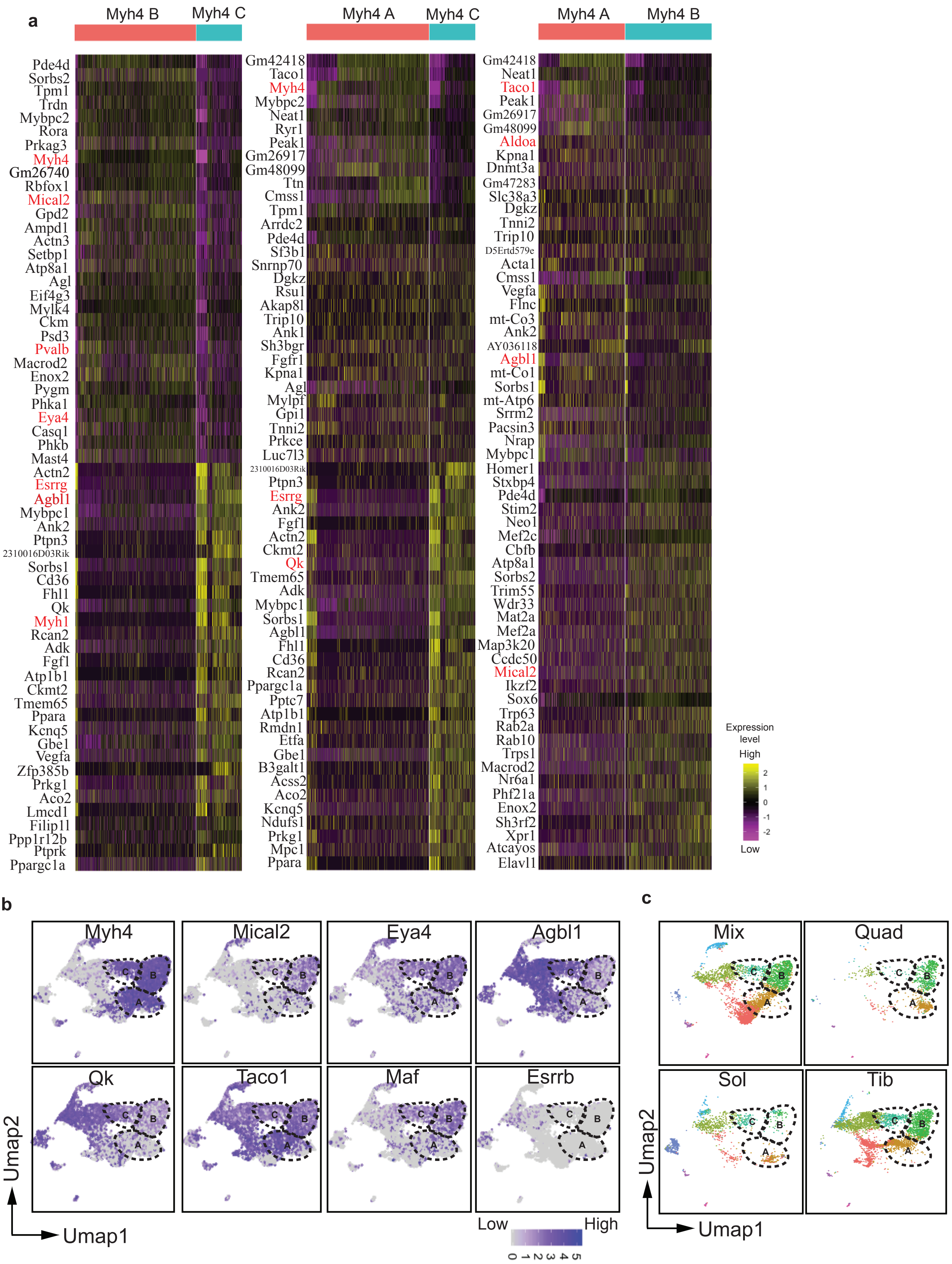

Supplementary Figure 3. The different populations of *Myh4*+ myonuclei. **a** Heatmap of genes upregulated (yellow) and downregulated (violet) in the different clusters of *Myh4* positive nuclei. The values correspond to z-scores of normalized counts. **b** Same Umap diagram than in Fig. 1c showing the expression of several genes differentially expressed in the 3 sub-clusters of *Myh4* myonuclei. The intensity of the violet color depends on the gene expression level. **c** Same Umap diagram than in Fig. 1c showing the origin of each nucleus.

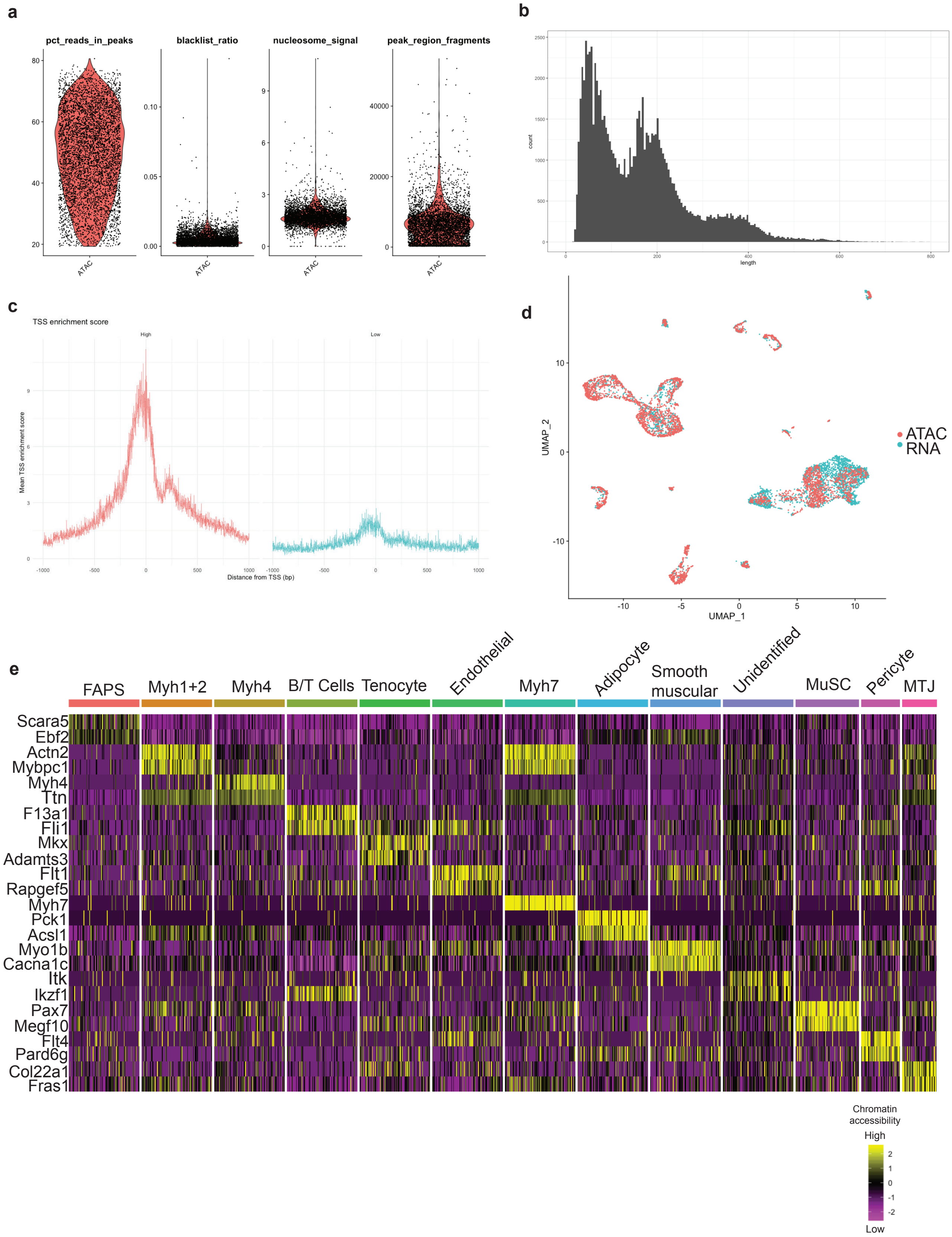

Supplementary Figure 4. Quality controls of the snATAC-seq experiments from Fig. 3. a Violin plot from snATAC-seq experiments from Fig. 3, showing the percentage of reads in peaks, the ratio of reads in blacklist region versus peaks region, the nucleosome signal and the peak region fragments. **b** Histogram showing the fragment length periodicity for all the nuclei from snATAC-seq experiments. **c** Histogram showing the enrichment of the peaks from snATAC-seq at transcriptional start sites (TSSs). **d** UMAP plot showing the co-embedding of snRNA-seq and snATAC-seq experiments from adult soleus and quadriceps. **e** Heatmap of the top two genes with preferentially chromatin accessibility (yellow) in each population of nuclei of snATAC-seq experiments. The values correspond to z-scores of normalized counts.

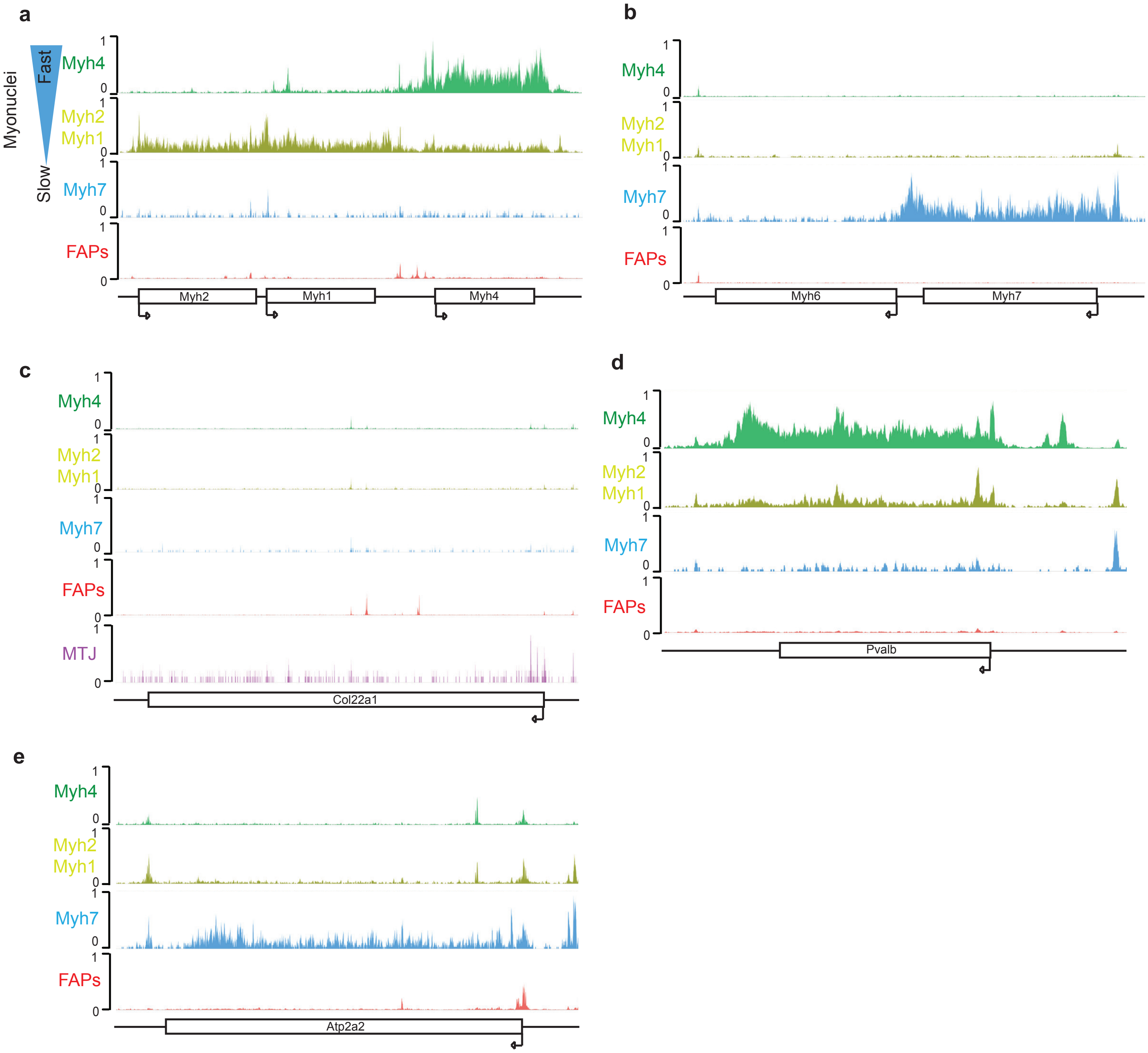

Supplementary Figure 5. Chromatin accessibility in adult myonuclei by snATAC-seq experiments. **a** Chromatin accessibility in the f*Myh* locus in different types of nuclei present in skeletal muscles. **b** Same as **a** for the slow *Myh7* locus. **c** Same as **a** for the *Col22a1* locus. **d** Same as **a** for the *Pvalb* locus. **e** Same as **a** for the *Atp2a2* locus.

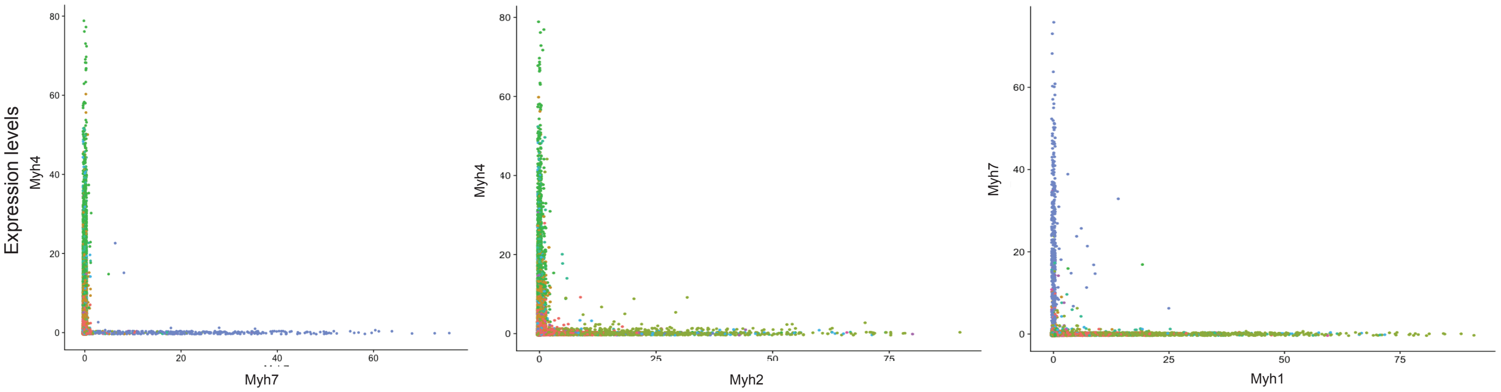

Supplementary Figure 6. The majority of myonuclei express only one isoform of *Myh*. Analysis of *Myh* isoforms expression in myonuclei from snRNA-seq data. Each dot corresponds to a myonucleus and the x- and y-axis corresponds to the indicated *Myh* expression level.

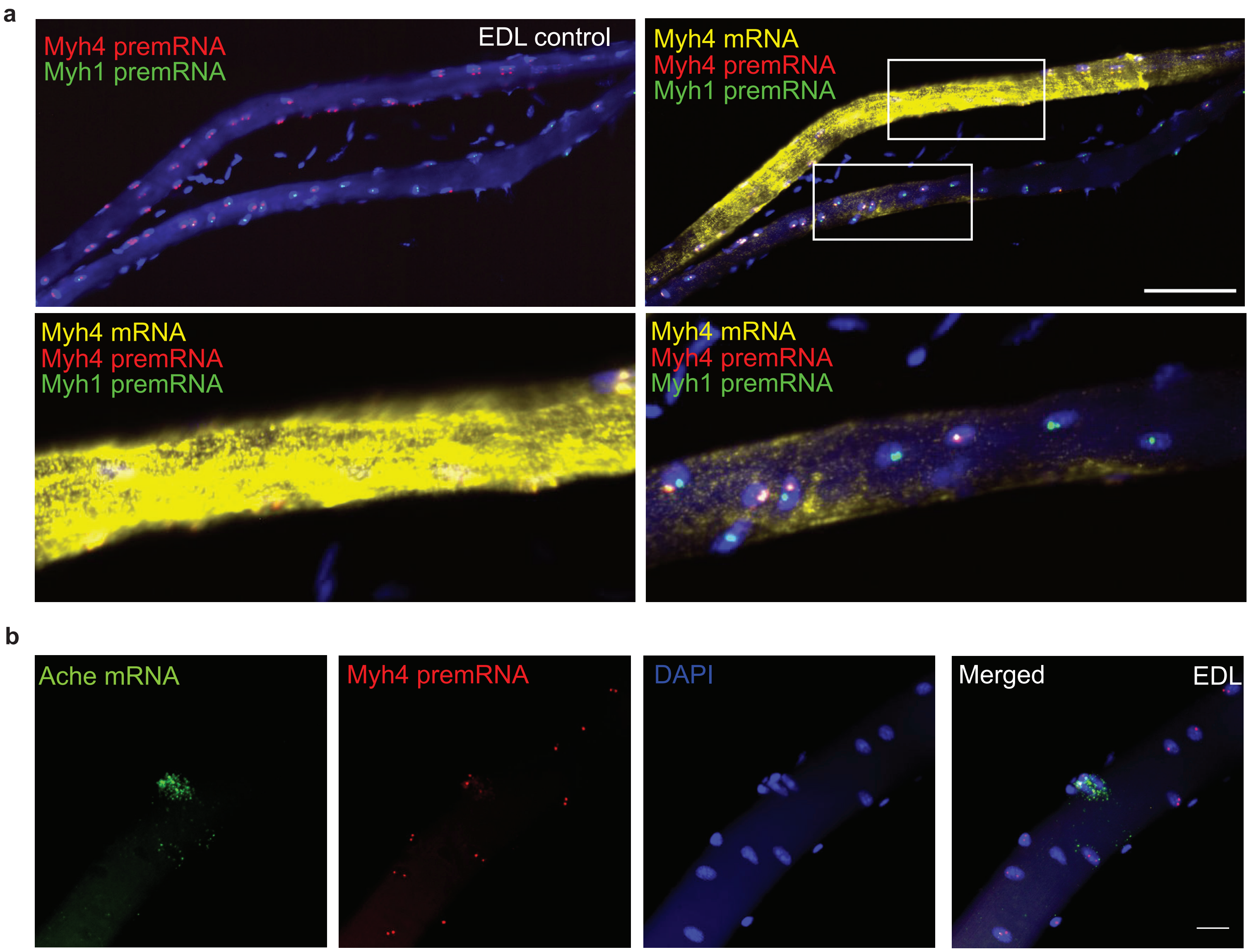

Supplementary Figure 7. Transcriptional variability of *Myh* expression all along myofibers. a Up: RNAscope on isolated fibers from EDL showing the localization of *Myh4* (red), *Myh1* (green) pre-mRNAs and *Myh4* (yellow) mRNAs in pure and hybrid fibers. Down left: the coordinated fiber shows a homogeneous accumulation of *Myh4* mRNA (in yellow) all along the myofiber. Down right: the hybrid fiber shows an accumulation of *Myh4* mRNA around *Myh4* positive nuclei (arrowhead) but not around *Myh1* positive nuclei. The number of hybrid fibers presenting non homogenous *Myh4* mRNA accumulation is very low in EDL. **b** RNAscope showing the expression of *Ache* mRNA and *Myh4* pre-mRNA in NMJ nuclei (arrowhead). For a scale bar: 100µm. For b scale bar: 20µm. For b representative RNAscope experiments are presented, N=3.

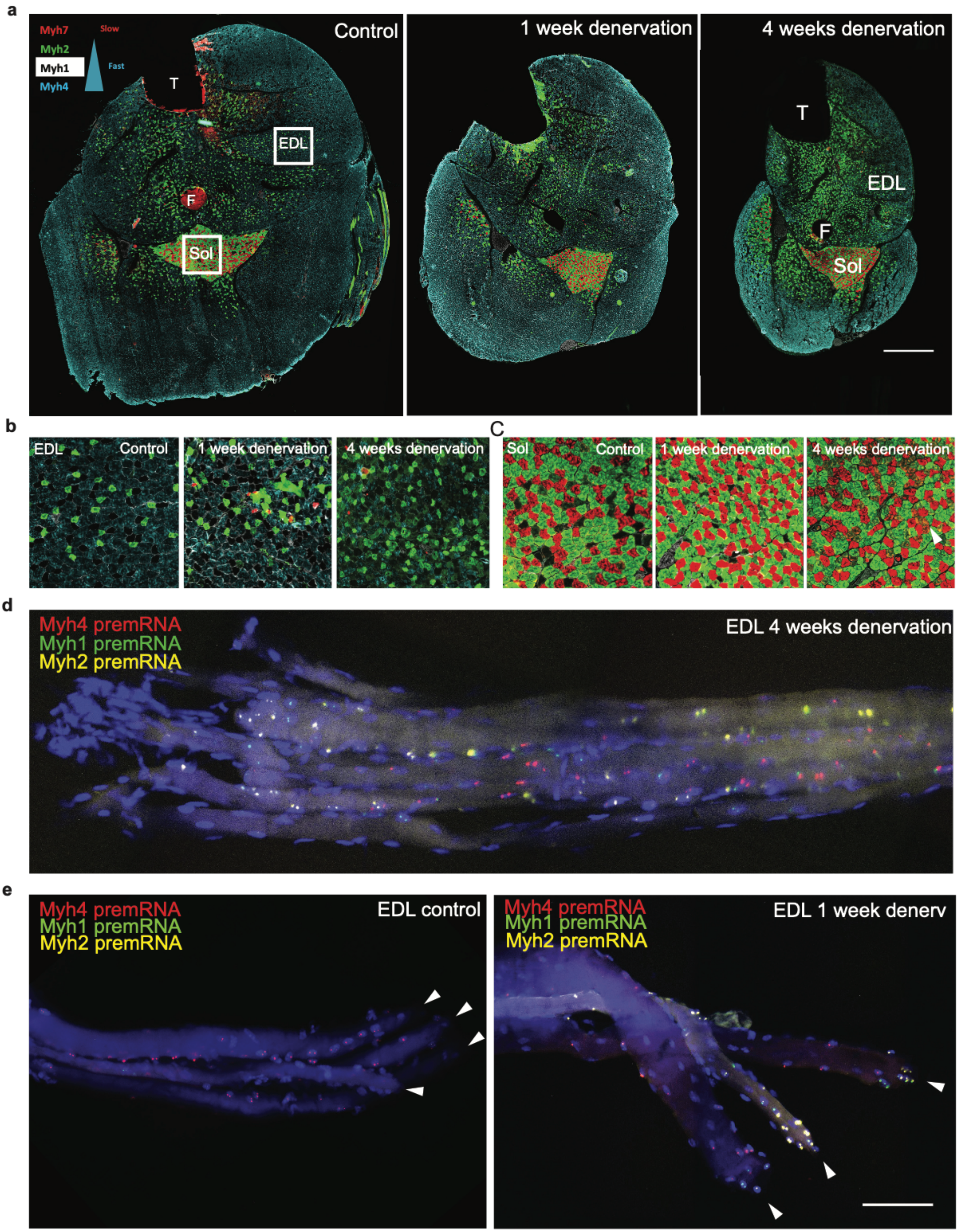
a
Myh7
Myh2
Myh1
Myh4
Slow
Fast
Control
T
EDL
F
Sol
1 week denervation
4 weeks denervation
b
EDL
Control
1 week denervation
4 weeks denervation
C
Sol
Control
1 week denervation
4 weeks denervation
d
Myh4 premRNA
Myh1 premRNA
Myh2 premRNA
EDL 4 weeks denervation
e
Myh4 premRNA
Myh1 premRNA
Myh2 premRNA
EDL control
EDL 1 week denerv

Supplementary Figure 8. *Myh* expression during denervation. a Immunostaining of MYH proteins in a section of the leg of an adult mouse after 1 week (center) and 4 weeks of denervation (right) and control (left). During denervation, fast MYH4 fibers located on the periphery of the leg show preferential atrophy compared to the slower fibers in the center of the leg. Fibers from all muscles become slower (fast to slow transition) : muscles have less MYH4 (blue) fibers and more MYH1 (black), MYH2 (green) and MYH7 (red) fibers. T: Tibia; F: Fibula; Sol: Soleus; EDL: Extensor digitalis longus. **b** Same as (**a**). Zoom in EDL muscle. **c** Same as (**a**). Zoom in soleus muscle. **d** RNAscope on isolated fibers from 4 weeks denervated EDL, showing the localization of *Myh4* (red), *Myh1* (green) and *Myh2* (yellow) pre- mRNAs. After 4 weeks of denervation, fibers are still de-coordinated. **e** RNAscope on isolated fibers from control and 1 week denervated EDL, showing the localization of *Myh4* (red), *Myh1* (green) and *Myh2* (yellow) pre-mRNAs. Left: Nuclei from the myotendinous junctions of control EDL fibers express the same *Myh* isoform than other nuclei (arrowhead). Right: After 1 week of denervation, MTJ nuclei (arrowhead) express different isoforms of *Myh* than the other myonuclei, and this expression seems more important than in the other nuclei. For a scale bar: 1000μm. For e scale bar: 100μm. For a-e, representative images are presented: N=3.

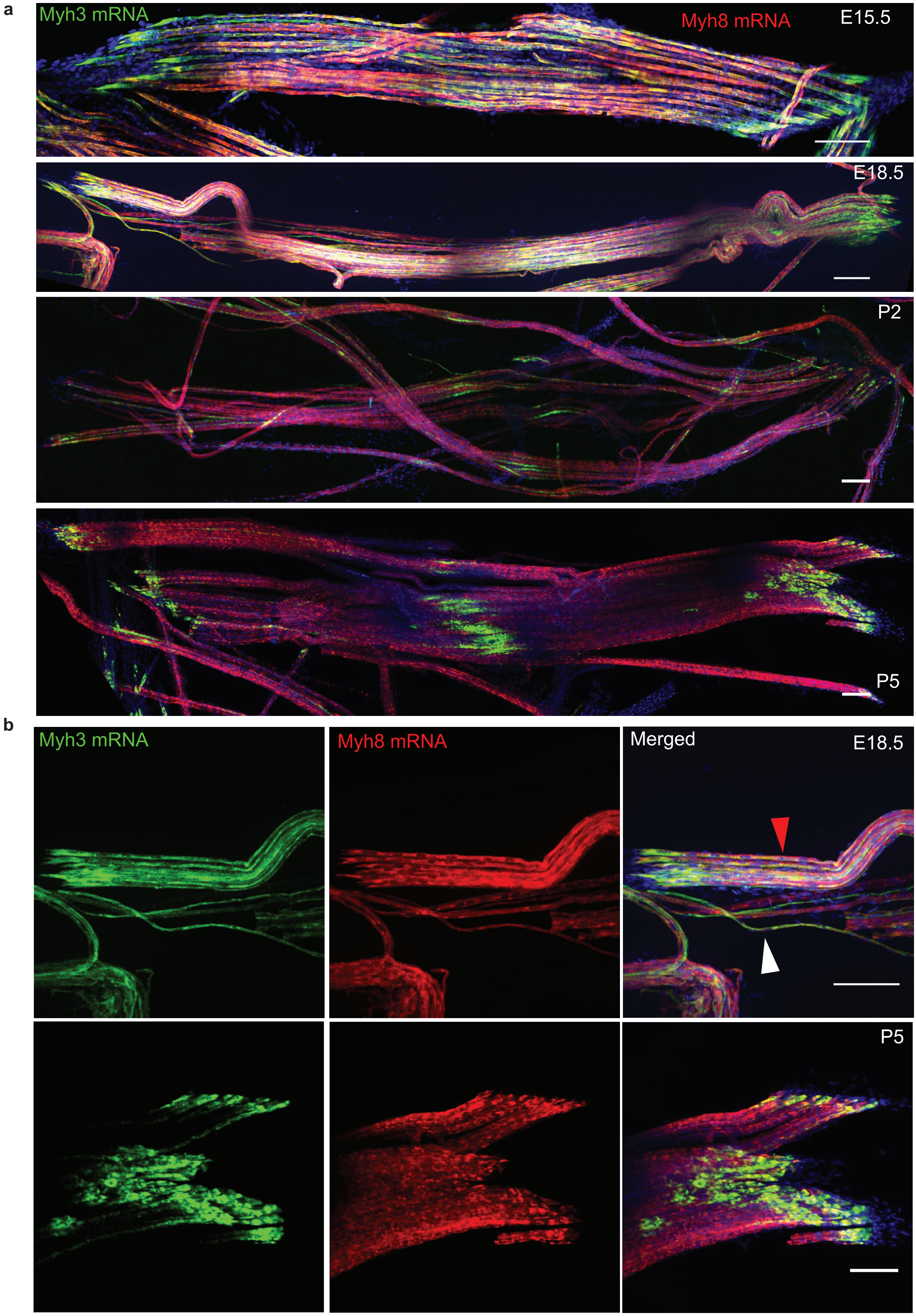
a
Myh3 mRNA
Myh8 mRNA
E15.5
E18.5
P2
P5
b
Myh3 mRNA
Myh8 mRNA
Merged
E18.5
P5

Supplementary Figure 9. *Myh3* expression during development is regionalized in MTJ nuclei in contrast to *Myh8* expression. a RNAscope against *Myh3* (green) and *Myh8* (red) mRNA on isolated fibers from forelimbs at E15.5, E18.5, P2 and P5. At E15,5 embryonic myofibers express *Myh3* and *Myh8*. The myotendinous junction areas show an accumulation of *Myh3* mRNA. At E18,5, fetal myofibers (small fibers) expressed more *Myh3* than embryonic fibers (big fibers). **b** Same as (**a**). Zoom in MTJ areas at E18,5, and P5. *Myh3* (green) accumulated in MTJ areas in contrast to *Myh8* (red). White arrowhead shows a primary myofiber and the red arrowhead a secondary myofiber. For a, b scale bar: 100µm. For a and b representative RNAscope experiments are presented, N=3.

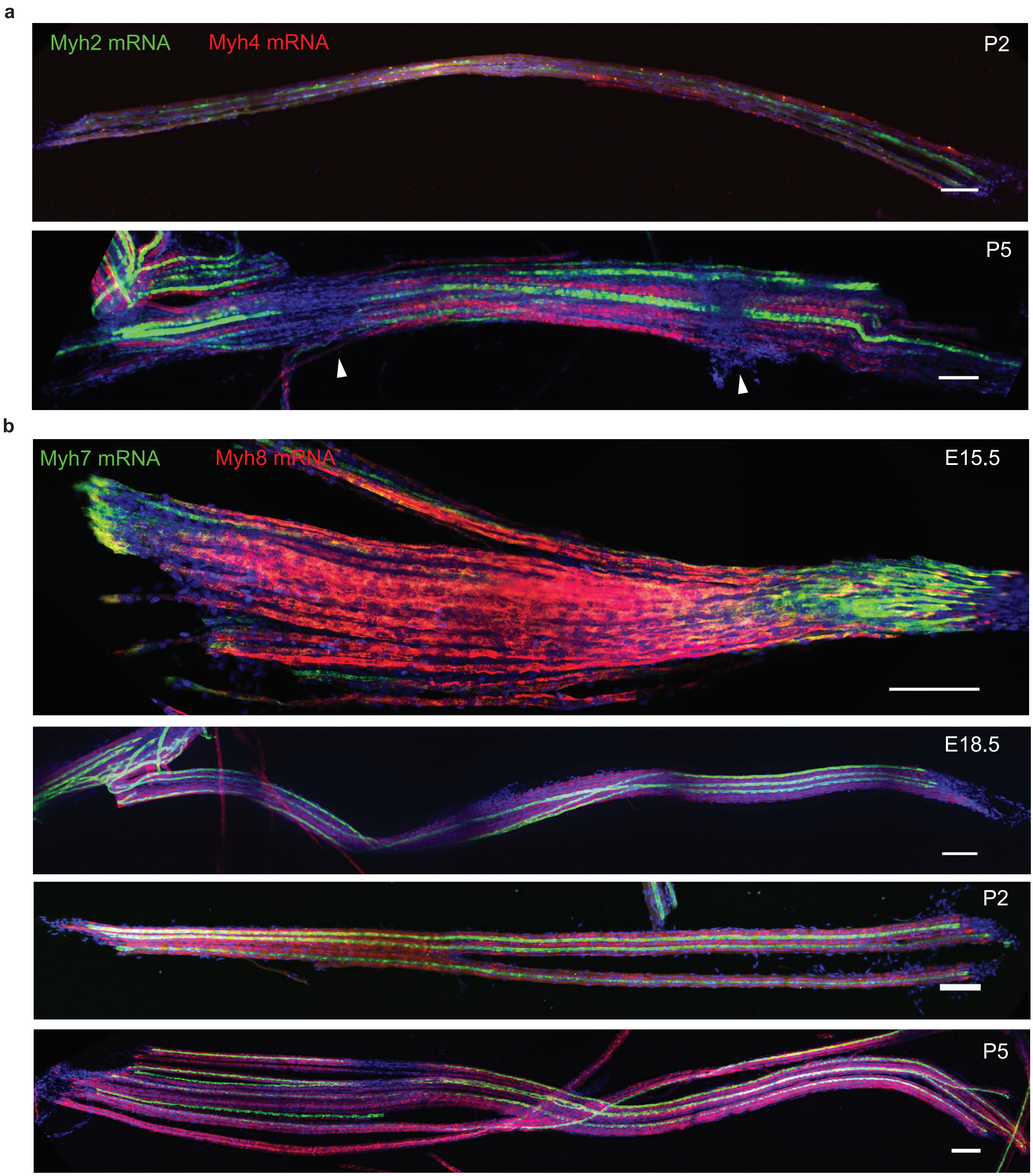

Supplementary Figure 10. *Myh7, Myh2 and Myh4* expression during development. a RNAscope against *Myh4* (red) and *Myh2* (green) mRNAs on isolated fibers at P2 and P5. The adult fast *Myh2* and *Myh4* mRNAs start to be detectable in some myofiber regions at P2. The expression is strongly increased at P5 and is localized homogeneously along the myofiber. We did not detect hybrid *Myh4* and *Myh2* myofibers. Note that some parts of the myofibers at P5 do not present *Myh2* nor *Myh4* mRNA accumulation (arrowhead). **b** RNAscope against *Myh7* (green) and *Myh8* (red) mRNAs on isolated fibers at E15.5, E18.5, P2 and P5. *Myh7* mRNAs accumulate in MTJ areas of all myofibers at E15.5. After E18.5, *Myh7* mRNAs are detected homogeneously in slow myofibers and no more accumulating in MTJ areas. For a, b scale bar: 100µm. For a and b representative RNAscope experiments are presented, N=3.

Target	Antibody reference	Suplier	Species	dilution
Myh7	BA-F8	DHSB	mouse IgG2B	1/40.
Myh2	SC-71	DHSB	mouse IgG1	1/200
Myh1	6H1	DHSB	mouse IgM	1/40.
Myh4	BF-F3	DHSB	mouse IgM	1/200
Laminin	L9393	Sigma	Rat	1/500

Supplementary Table 1. List of antibodies used in the study.
