# Supplementary material for: Single-nucleus RNA-seq and FISH identify coordinated transcriptional activity in mammalian myofibers
Source: Nat Commun. 2020 Oct 9;11:5102. doi: 10.1038/s41467-020-18789-8 (PMC7547110; doi:10.1038/s41467-020-18789-8)
Supplement: Supplementary file 3 — Description of Additional Supplementary Files [file 41467_2020_18789_MOESM3_ESM.pdf]

Title: Supplementary Data 1.

Description: Table of differently expressed genes between clusters from snRNAseq data
