# Supplementary material for: Single-nucleus RNA-seq and FISH identify coordinated transcriptional activity in mammalian myofibers
Source: Nat Commun. 2020 Oct 9;11:5102. doi: 10.1038/s41467-020-18789-8 (PMC7547110; doi:10.1038/s41467-020-18789-8)
Supplement: Supplementary file 4 — Reporting Summary [file 41467_2020_18789_MOESM4_ESM.pdf]

nature research

Corresponding author(s): Pascal Maire

Last updated by author(s): Aug 27, 2020

Reporting Summary

Nature Research wishes to improve the reproducibility of the work that we publish. This form provides structure for consistency and transparency in reporting. For further information on Nature Research policies, see our Editorial Policies and the Editorial Policy Checklist.

Statistics

For all statistical analyses, confirm that the following items are present in the figure legend, table legend, main text, or Methods section.

n/a	Confirmed	
☐	☒	The exact sample size (n) for each experimental group/condition, given as a discrete number and unit of measurement
☐	☒	A statement on whether measurements were taken from distinct samples or whether the same sample was measured repeatedly
☐	☒	The statistical test(s) used AND whether they are one- or two-sided<br>*Only common tests should be described solely by name; describe more complex techniques in the Methods section.*
☐	☒	A description of all covariates tested
☒	☐	A description of any assumptions or corrections, such as tests of normality and adjustment for multiple comparisons
☐	☒	A full description of the statistical parameters including central tendency (e.g. means) or other basic estimates (e.g. regression coefficient) AND variation (e.g. standard deviation) or associated estimates of uncertainty (e.g. confidence intervals)
☐	☒	For null hypothesis testing, the test statistic (e.g. F, t, r) with confidence intervals, effect sizes, degrees of freedom and P value noted<br>*Give P values as exact values whenever suitable.*
☒	☐	For Bayesian analysis, information on the choice of priors and Markov chain Monte Carlo settings
☒	☐	For hierarchical and complex designs, identification of the appropriate level for tests and full reporting of outcomes
☒	☐	Estimates of effect sizes (e.g. Cohen's d, Pearson's r), indicating how they were calculated

Our web collection on statistics for biologists contains articles on many of the points above.

Software and code

Policy information about availability of computer code

Data collection	BD FACSDIVA software 8.0.3,MetaMorph 7.7.5
Data analysis	Cell Ranger Single Cell Software Suite 3.0.2, R (v 3.4.3), Seurat (v3.0.2), Signac (v0.2.4), Chromvar (v1.1.1), Fiji (ImageJ 2.0.0), Graphpad (v6.0)

For manuscripts utilizing custom algorithms or software that are central to the research but not yet described in published literature, software must be made available to editors and reviewers. We strongly encourage code deposition in a community repository (e.g. GitHub). See the Nature Research guidelines for submitting code & software for further information.

Data

Policy information about availability of data

All manuscripts must include a data availability statement. This statement should provide the following information, where applicable:

- Accession codes, unique identifiers, or web links for publicly available datasets
- A list of figures that have associated raw data
- A description of any restrictions on data availability

Single nucleus RNA-seq and Single nucleus ATAC-seq data are available in the NCBI Gene Expression Omnibus (GEO) database under accession GSE150065

Field-specific reporting

Life sciences study design

All studies must disclose on these points even when the disclosure is negative.

Sample size	No statistical method was used to predetermine sample size. For snRNA-seq on the 10x genomics system, 15 167 nuclei were collected that had passed quality filtering. For snATAC-seq, we used 6500 nuclei that had passed quality filtering. The number of nuclei encapsulated in the 10x genomic machine has been determined according to the parameters recommended by the company 10x to reduce the number of doublet of nuclei encapsulated.For RNAscope experiments we used three or more animals per condition, and counted a minimum of 50 fibers. Significant differences between mean values were evaluated using two-way ANOVA with Graphpad 6 software and student t test.
Data exclusions	The snRNA-seq and snATAC-seq samples of low quality were excluded as explained in the "Methods" section . Essentially, we excluded nuclei that had few reads or few associated features.
Replication	For snRNA-seq and snATAC-seq, data from thousand of cells were collected for each condition, providing replicate measurements for all different conditions/cell sub-populations. For FISH experiments, three biological replicates were used for each condition.
Randomization	Experiments have been performed randomly.
Blinding	The investigators were not blinded in regard to allocation of samples during experiments and outcome assessment. For snRNA-seq and snATAC-seq analysis, unsupervised machine learning was applied, and no prior knowledge was used to determine different clusters.

Reporting for specific materials, systems and methods

We require information from authors about some types of materials, experimental systems and methods used in many studies. Here, indicate whether each material, system or method listed is relevant to your study. If you are not sure if a list item applies to your research, read the appropriate section before selecting a response.

Materials & experimental systems

n/a	Involved in the study	
☐	☒	Antibodies
☒	☐	Eukaryotic cell lines
☒	☐	Palaeontology and archaeology
☐	☒	Animals and other organisms
☒	☐	Human research participants
☒	☐	Clinical data
☒	☐	Dual use research of concern

Methods

n/a	Involved in the study	
☒	☐	ChIP-seq
☒	☐	Flow cytometry
☒	☐	MRI-based neuroimaging

Antibodies

Antibodies used	Target Antibody reference Suplier Species dilution<br>Myh7 BA-F8 DHSB mouse IgG2B 1/40.<br>Myh2 SC-71 DHSB mouse IgG1 1/200<br>Myh1 6H1 DHSB mouse IgM 1/40.<br>Myh4 BF-F3 DHSB mouse IgM 1/200<br>Laminin L9393 Sigma Rat 1/500
Validation	The antibodies have been validated by several studies as specified by:<br>PMID: 22530000 for Myh antibodies<br>PMID: 29733324 for Laminin antibodies

Animals and other organisms

Policy information about studies involving animals; ARRIVE guidelines recommended for reporting animal research

Laboratory animals	6-8 weeks old C57bl6N females were used in this study. Mice were maintained at temperature 22+/-2 °C, with 30 to 70% humidity and with a dark/light cycle of 12h/12H.
Wild animals	No wild type animals were used
Field-collected samples	No field collected samples
Ethics oversight	Animal experimentations were carried out in strict accordance with the European STE 123 and the French national charter on the Ethics of Animal Experimentation. Protocols were approved by the Ethical Committee of Animal Experiments of the Institut Cochin, CNRS UMR 8104, INSERM U1016, and by the Ministère de l'éducation nationale, de l'enseignement et de la recherche, n° APAFIS#15699-2018021516569195.

Note that full information on the approval of the study protocol must also be provided in the manuscript.
